# Mining Small Molecules from *Teredinibacter turnerae* Strains Isolated from Philippine Teredinidae

**DOI:** 10.3390/metabo12111152

**Published:** 2022-11-21

**Authors:** Jamaine B. Villacorta, Camille V. Rodriguez, Jacquelyn E. Peran, Jeremiah D. Batucan, Gisela P. Concepcion, Lilibeth A. Salvador-Reyes, Hiyas A. Junio

**Affiliations:** 1 Small Molecules Profiling Laboratory (SMPL), Institute of Chemistry, College of Science, University of the Philippines, Diliman, Quezon City 1101, Philippines; 2 Marine Science Institute, College of Science, University of the Philippines, Diliman, Quezon City 1101, Philippines

**Keywords:** Philippine shipworm symbionts, molecular networking, GNPS, marine metabolomics, *Teredinibacter turnerae*

## Abstract

Endosymbiotic relationship has played a significant role in the evolution of marine species, allowing for the development of biochemical machinery for the synthesis of diverse metabolites. In this work, we explore the chemical space of exogenous compounds from shipworm endosymbionts using LC-MS-based metabolomics. Priority *T. turnerae* strains (1022X.S.1B.7A, 991H.S.0A.06B, 1675L.S.0A.01) that displayed antimicrobial activity, isolated from shipworms collected from several sites in the Philippines were cultured, and fractionated extracts were subjected for profiling using ultrahigh-performance liquid chromatography with high-resolution mass spectrometry quadrupole time-of-flight mass analyzer (UHPLC-HRMS QTOF). *T. turnerae* T7901 was used as a reference microorganism for dereplication analysis. Tandem MS data were analyzed through the Global Natural Products Social (GNPS) molecular networking, which resulted to 93 clusters with more than two nodes, leading to four putatively annotated clusters: lipids, lysophosphatidylethanolamines, cyclic dipeptides, and rhamnolipids. Additional clusters were also annotated through molecular networking with cross-reference to previous publications. Tartrolon D cluster with analogues, turnercyclamycins A and B; teredinibactin A, dechloroteredinibactin, and two other possible teredinibactin analogues; and oxylipin (E)-11-oxooctadec-12-enoic acid were putatively identified as described. Molecular networking also revealed two additional metabolite clusters, annotated as lyso-ornithine lipids and polyethers. Manual fragmentation analysis corroborated the putative identification generated from GNPS. However, some of the clusters remained unclassified due to the limited structural information on marine natural products in the public database. The result of this study, nonetheless, showed the diversity in the chemical space occupied by shipworm endosymbionts. This study also affirms the use of bioinformatics, molecular networking, and fragmentation mechanisms analysis as tools for the dereplication of high-throughput data to aid the prioritization of strains for further analysis.

## 1. Introduction

The Philippines, identified to be one of the 17 megadiverse countries [1], harbor extensive marine and terrestrial species. One of the interesting marine organisms found in the Philippines are shipworms or tamilok. A rare giant shipworm (*Kuphus polythalamia*) discovered in Sultan Kudarat, Maguindanao contains bacterial symbionts that feed on hydrogen sulfide instead of cellulose [2].

*Teredinibacter turnerae* is one of the most predominant shipworm symbionts investigated. It is a Gram-negative proteobacterium that has been isolated from the gills of wood-boring shipworms [3]. *T. turnerae* T7901 has been found to contain complex polyketide synthase and nonribosomal peptide synthetase biosynthetic gene clusters that can possibly be a source of potentially bioactive compounds [4,5]. As of late, four classes of compounds were purified from *T. turnerae* T7901. Turnerbactin, a siderophore structurally similar to catecholate siderophores trivanchrobactin and trychrysobactin, is hypothesized to play a role in keeping the gut of shipworms microbe-free [6]. Macrodiolide polyketides with antibacterial properties, tartrolon D, and its boronated counterpart tartrolon E, were reported to inhibit other shipworm symbionts and pathogenic bacteria. In addition, the shipworm host can maximize the uptake of glucose liberated by the breakdown of wood using these compounds [7]. Turnercyclamycins A and B are lipopeptide antibiotics potent against Gram-negative pathogens *Escherichia coli*, *Klebsiella pneumoniae*, and the multi-drug resistant *Acinetobacter baumannii* [8]. Lastly, teredinibactins, a class of metal-binding compounds with a phenolate-thiazoline moiety that coordinates metal ions were also purified from *T. turnerae* [9]. In the paper by Yang et al., [4] genomic analysis of *T. turnerae* strain T7901 showed a large proportion of its genome dedicated to functions in secondary metabolism. The combined putative secondary metabolites pathways account for 7% of the *T. turnerae* genome which is comparable *to Streptomyces coelicolor* [10] and *Streptomyces avermitilis* [11]. This significant devotion of *T. turnerae* in secondary metabolism suggests a potential for natural product discovery.

Metabolomics deals with the large-scale analysis of small molecules in a given organism [12]. It allows the investigation of complex mixtures, e.g., extracts from microbial culture, leading to the putative identification of small molecules without the need for prior isolation of active compounds [13]. Potential bioactivity can also be related to the presence of certain metabolites. This method helps to make natural product discovery less tedious than conventional ones. Current metabolomics on *T. turnerae* is limited to the identification of the aryl-homoserine lactone (AHL) [14]. However, metagenomics projected a considerable number of small molecules based on the biosynthetic gene cluster annotations [5]. While there are many studies on the genomics and biosynthetic potential of *T. turnerae*, there is limited information on the metabolome from this organism. Thus, metabolite mining of Teredinidae endosymbionts is undertaken to explore the chemical space of these marine microorganisms using metabolomics. *T. turnerae* strains (1022X.S.1B.7A, 991H.S.0A.06B, 1675L.S.0A.01) isolated from shipworms collected from different locations in the Philippines and the reference *T. turnerae* strain, T7901, were profiled and compared for dereplication. The three (3) strains (1022X.S.1B.7A, 991H.S.0A.06B, and 1675L.S.0A.01) showed potent inhibition of microbial pathogens, which made them the three priority strains to be analyzed. GNPS molecular networking showed considerable diversity of profiled compounds.

## 2. Materials and Methods

### 2.1. Shipworm Collection, Symbionts Isolation, and Processing

Collection of shipworm specimens and purification of *T. turnerae* were carried out by the Philippine Mollusk Symbiont-International Cooperative Biodiversity Group in 2010 under the Gratuitous Permit #FBP-0036-10 issued by the Department of Agriculture. The purified microbial isolates were stored in 20% (*v*/*v*) cryoprotectant glycerol (Thermo Fisher Scientific, Japan) and stored at −75 °C, as part of the Marine Natural Products Legacy Collection at the Marine Science Institute. Table 1 shows the four (4) isolates selected as a priority for analysis.

Each microbial isolate was revived and cultivated in SBM + 0.2% Sigmacell cellulose Type 101 agar plate at 30 °C according to the method of Waterbury et al. [3] and Distel et al. [15]. One colony was transferred to 100 mL SBM broth for 3 to 5 days at 30 °C, 150 rpm. Briefly, 5 mL inocula were transferred to a freshly prepared 1L SBM. Culture flasks were incubated for 7 days at 30 °C and 150 rpm. The broth suspension was centrifuged at 4000 rpm for 20 min. Diaion HP20 (Supelco, Sigma-Aldrich, St. Louis MO, USA) was added to the collected supernatant for at least 2 h, followed by subsequent washing of distilled water and 25% MeOH: H_2_O (RCI Labscan, Bangkok, Thailand). The 100% methanol extracts were collected and dried in vacuo to yield the crude extracts.

### 2.2. Fractionation of the Crude Methanol Extracts

Methanol (100%) crude extracts were partitioned with ethyl acetate:H_2_O (1:1 *v*/*v*, 3×), and the organic extracts were collected and then dried in vacuo. For *T. turnerae* 991H.S.0A.06B and 1675L.S.0A.01, the ethyl acetate extracts were subjected to a pressure-assisted open column chromatography using C_18_ resin (40 mm × 60 mm) stationary phase, and stepwise gradient elution of MeOH in H_2_O at 20% increment. The collected 100% MeOH open column fraction (OCF) which showed activity was further purified with Reversed-Phase High-Performance Liquid Chromatography (RP-HPLC) using a semipreparative Phenomenex Luna C_8_ column (250 × 10 mm, 4 µm) with a linear gradient of 40–100% CH_3_CN/H_2_O for 10 min, followed by 100% CH_3_CN for 10 min, at 2.5 mL/min flow rate. 

For *T. turnerae* 1022X.S.1B.7A, the ethyl acetate extract was purified via open column chromatography using Sephadex^®^ LH-20 (10 mm × 160 mm) resin, and isocratic elution of MeOH to collect a total of seven fractions. The LH-20 fractions 2 and 3 which showed activity was further purified with RP-HPLC using a semipreparative Phenomenex Luna C_8_ column (250 × 10 mm, 4 µm) with a linear gradient of 40–100% CH_3_CN/H_2_O for 10 min, followed by 100% CH_3_CN for 10 min, at 2.5 mL/min flow rate. The LH-20 fractions 5 and 6 which were not active but showed the possible presence of teredinibactins were further purified with RP-HPLC using a semipreparative Synergi-Hydro^®^ C_18_ column (250 × 10 mm, 4 µm) with a linear gradient of 40–100% CH_3_CN/H_2_O (both with 0.1% TFA) for 10 min, followed by 100% CH_3_CN (with 0.1% TFA) for 5 min, at 2.5 mL/min flow rate. The bioactivity profile of fractions of different *T. turnerae* strains is illustrated in Figure S1.

### 2.3. LC-MS Profiling and Dereplication of T. turnerae Strains

Vacuum-dried methanolic extracts and fractions from HPLC and open column chromatography of *T. turnerae* strains were resuspended in 50% (*v*/*v*) LC-MS grade acetonitrile (Merck LiChrosolv^®^, Burlington, MA, USA) to a final concentration of 1.0 mg/mL for analysis. Untargeted metabolomics of exogenous metabolites of shipworm isolates was performed using Waters Acquity UPLC^®^ H-Class System with an ESI Xevo^®^ G2-XS Quadrupole Time-of-Flight (QToF) mass analyzer with an electrospray ionization (ESI) source. A 0.5 µL sample injection volume into an Acquity UPLC CSH C_18_ Column (1.7 µm, 50 mm long, 2.1 mm I.D.) was maintained at 30 °C. Acetonitrile (B) and water (A) both infused with 0.1% formic acid (Thermo Scientific, Rockford, IL, USA) were used as mobile phase. The flow rate was at a constant rate of 0.350 mL/min. Gradient elution is as follows: 5% B at 0.0 to 1.0 min, 5–100% B at 1 to 10.0 min, 100% B at 10.0 to 12.0 min, 100% to 5%B at 12.0–13.0 min, and then re-equilibrating back to 5% B at 13.0 to 15.0 min. 

ESI in the positive ionization mode was carried out with the following settings: capillary voltage at 3.0 kV, cone voltage at 42 kV, and source offset of 80 kV. The source temperature was maintained at 150.0 °C, and the desolvation gas temperature was at 500 °C. Acquisition of full scans (MS^1^) was done at a mass range of *m/z* 50.0 to 1500.0, and with a scan time of 0.50 s. MS^2^ analysis was performed using fast data-dependent acquisition (DDA) mode for ions with intensity exceeding 3.0 × 10^5^ thresholds. A maximum of the eight most abundant ions per scan were selected for MS^2^ analyses. Precursor ions were subjected to collision-induced dissociation (CID) with argon curtain under fixed collision energy of 15.0 eV as well as collision energy ramps of 15.0–30.0 eV, 30.0–45.0 eV, and 45.0–60.0 eV. 

### 2.4. Data Processing

Data gathered from MS^2^ analysis were analyzed through the cloud-based bioinformatic platform Global Natural Products Social Molecular Networking (GNPS) [16] for library matching and molecular networking. Parameters for library matching were set as follows: precursor ion mass tolerance of *m/z* 0.02, fragment ion mass tolerance of *m/z* 0.02, a minimum cosine (similarity) score of 0.70, and minimum matched peaks of 6. MSCluster was activated for the creation of molecular networks. Parameters used for the generation of molecular networks include a minimum similarity (cosine) of 0.70 calculated from the fragmentation pattern of two precursor ions, six matched peaks, and a maximum number (top K) of five neighbors. Molecular networks were visualized and customized using Cytoscape 3.7.1 [17].

## 3. Results and Discussion

**Figure 1:**
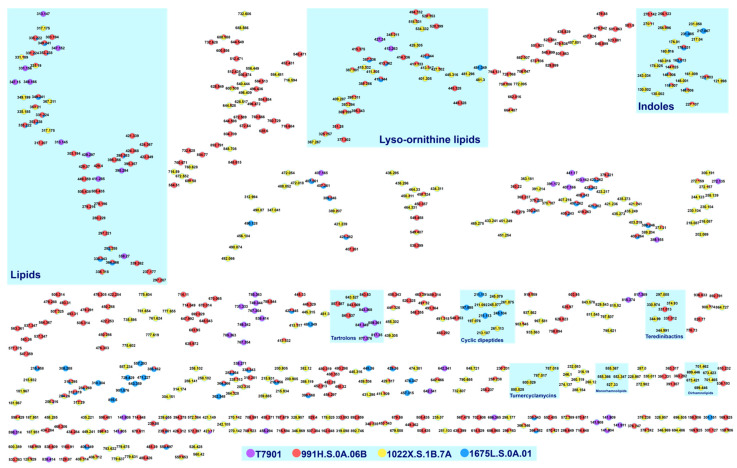
Molecular network of 35 samples consisting of crude, fractions, and subfractions of different *T. turnerae* strains, T7901, 991H.S.0A.06B, 1022X.S.1B.7A, and 1675L.S.0A.01 (Table S1). A total of 590 nodes (out of 1158 nodes) formed 93 clusters with at least one connection in the molecular network after removing self-looping nodes. Purple nodes represent compounds from the seed isolate, T7901, and compounds from 991H.S.0A.06B (red), 1022X.S.1B.7A (yellow), and 1675L.S.0A.01 (blue) marked accordingly.

**Figure 2:**
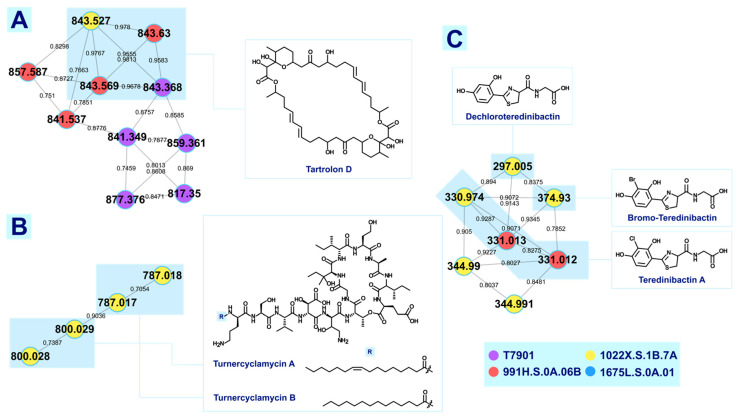
Clusters annotated through dereplication based on previous studies on *Teredinibacter* sp. Cluster (**A**) consisting of 10 nodes was putatively identified as tartrolon D and its possible analogues. Multiple nodes of *m/z* 843 can be seen in this cluster partly due to different collision energy used for the data-dependent acquisition of MS^2^ and the precursor ion mass tolerance set by the user in generating the molecular network. The same phenomenon was observed in cluster (**B**) putatively identified as turnercyclamycins cluster, where repeating nodes are due to the different collision energies used in the method for data-dependent acquisition (DDA). Cluster (**C**) was putatively identified as teredinibactins where nodes *m/z* 330.974, 331.013, and 331.012 were identified as teredinibactin A, and 297.005 was identified as decholoroteredinibactin. Analogues of teredinibactin A were putatively identified in the cluster with *m/z* 374.930 as the boronated counterpart of teredinibactin A, and 344.991 as teredinibactin A with an extra methyl group. Nodes highlighted in light blue are considered the same compound.

The GNPS platform allows for the annotation of small molecules in complex mixtures using open-access spectral library search and molecular networking. Publicly curated spectral libraries are searched against sample MS/MS data for a possible compound match. Meanwhile, compounds with similar structural motifs producing similar fragmentation patterns are visually represented as clusters through molecular networks. These open-source chemoinformatic tools are useful in mining the metabolome of the highly diverse microenvironment of shipworm endosymbionts. Although there are a few caveats in using GNPS, which can affect the matching of compounds, i.e., limited database, MS^2^ spectra were acquired using different mass spectrometers and sample preparation methods [18], and cosine score algorithm [19], it has been shown to be helpful in assessing the chemical space of samples. Aside from solely relying on GNPS, dereplication based on previously identified compounds on *T. turnerae* was also employed for annotation. Reported MS/MS spectra of the compounds were compared against experimental data, which were corroborated with rational fragmentation pathways to putatively identify nodes forming a cluster. Lastly, manual annotation was used to label clusters with no GNPS spectral match and no prior literature report. Publicly available databases such as the Dictionary of Natural Products^®^ (DNP) and the Comprehensive Marine Natural Products Database (CMNPD) [20] were explored for compounds with similar mass to the node being analyzed. Therefore, accurate mass and fragmentation schemes were central to the putative identifications of features that fall under this category. Herein, we describe the profiling and annotation of exogenous metabolites from three priority *T. turnerae* strains (1022X.S.1B.7A, 991H.S.0A.06B, 1675L.S.0A.01) and reference strain (T7901) using the methods described above. Crude extracts of the *T. turnerae* priority strains and T7901 formed the molecular network shown in Figure 1. Of the 93 total clusters, only nine (9) clusters were annotated, namely, fatty acid amides, dipeptides, rhamnolipids, phosphatidylethanolamines, tartrolons, turnercyclamycins, teredinibactins, lyso-ornithine lipids (LOLs), and macrolides (Figure 2).

### 3.1. Dereplication Based on Previous Literature Studies on T. turnerae

#### 3.1.1. Tartrolons

Tartrolon D was first reported from the marine-derived actinomycete *Streptomyces* sp. MDG-04-17-069 as a cytotoxic macrodiolide [21]. Using high-resolution mass spectrometry (QSTAR Applied biosystems spectrometer and Agilent 1100 series LC/MSD spectrometer), Pérez et al. identified *m/z* 843.449 as tartrolon D with sodium adduct [21]. Antibacterial compounds tartrolon D and its boronated derivative tartrolon were previously isolated and identified through bioassay-guided fractionation of the crude extract of *T. turnerae* T7901 [7]. It was also mentioned by Altamia et al. [5] that GCF 11 for tartrolon was shared among several *T. turnerae* containing shipworms. Based on this information, a cluster consisting of ten (10) nodes with precursor ions ranging from *m/z* 817.350 to *m/z* 877.376 shown in Figure 2 was designated as the tartrolon cluster in the absence of GNPS spectral match. The same compound identity was also assumed based on the identical product ions generated from the accurate parent mass and reported in the literature. A representative sample with precursor ion [M+Na]^+^ (*m/z* 843.451, 1.19 ppm) putatively identified as tartrolon D was corroborated through manual fragmentation analysis (Figure S2). The majority of the product ions were formed through retro-heteroene reactions such as McLafferty-type rearrangements and retro-aldol fragmentations, which are common reactions observed in carbonyl-containing natural products [22].

In the tartrolon D cluster in Figure 2A, four repeating nodes of *m/z* 843 can be observed with a <0.1 Da difference in the representative mass. Inspection of product ions corresponding to each node showed identical fragmentation patterns consistent with tartrolon D based on published literature [7]. There are, however, minor differences in the relative intensities and accurate mass of product ions across samples, which is attributed to the complexity of samples and instrument conditions. Closer inspection revealed that the representative spectra reported by GNPS via MSCluster are not from the precursor ion with the highest intensity. MSCluster algorithm, which designates a representative spectrum, generates a “consensus spectrum” [23]. A consensus spectrum is achieved by summing the intensities of all the peaks across a mass axis [24]. 

MSCluster depends on the similarity threshold used to have the same spectra combined [23]. Three important inputs are needed in this algorithm; (1) the data elements or the spectra; (2) the similarity threshold and (3) the number of rounds the algorithm will compare a certain spectrum vs another spectrum. The algorithm doesn’t necessarily merge clusters with maximum similarity but joins the first ones it encounters that are within the similarity threshold provided by the user [23]. Having a finite number of rounds of comparison enables the algorithm to reduce the number of spectral similarity computations, saving computational costs [23]. However, a commonly reported problem with the use of MSCluster is the “fragmented clusters” or in the case of GNPS molecular networking, repeating nodes forming a connection in one cluster. This is intrinsic to the algorithm as it was designed with a certain limitation to save on computational costs [23]. This accounts for repeating nodes in the cluster having identical fragmentation patterns generated by the same compound. 

A cluster of tartrolon D analogues was putatively identified based on the manual inspection of the spectra of nodes corresponding to *m/z* 857.587, 859.361, and 841.349. A mass difference of 14 Da (857) and 16 Da (859) from the sodiated precursor ion of tartrolon D (843) suggested a methyl (-CH_3_) and a hydroxy (-OH) modification, respectively. Fragmentation patterns of the analogues can be accounted for by the proposed mechanism (Figure S2). Product ions from MS^2^ corroborate the modification at the R_1_ position by comparing *m/z* 389.22 vs 403.24 for the methylated analogue, and *m/z* 389.22 vs. 405.22 for the hydroxylated analogues (Figure S3). These putative analogues are significantly less abundant than tartrolon D. Another precursor ion (841) is suggested to have an extra unsaturation in the tartrolon D structure, with its position currently undetermined. A highly intense product ion peak (389) was observed to be common in the MS^2^ spectra of the compounds in this cluster. The structure is as shown (Figure S3) representing a monomer of tartrolon D.

#### 3.1.2. Turnercyclamycins

The biosynthetic pathway for turnercyclamycins is highly conserved in all shipworm isolates and gills [8]. These lipopeptide antibiotics, however, were not initially characterized in T7901 crude extract because of insolubility in commonly used solvents in the purification process [8]. Turnercyclamycins seem to be preferentially extracted in the insoluble boundary between the aqueous and ethyl acetate layers but are considerably stable once isolated [8]. 

A cluster composed of four nodes with precursor masses ranging from *m/z* 787 to 800 (Figure 2B) is annotated as turnercyclamycins. Experimental mass spectra for the precursor ions were inspected and compared to the reference mass spectra reported from HRESIMS for turnercyclamycins A and B [8]. Though there were slight differences in the relative abundances of the product ions due to dissimilar instrument parameters, the comparison was considered a positive match. Thus, turnercyclamycin A was putatively annotated with *m/z* 786.956 (3.18 ppm), and turnercyclamycin B with *m/z* 799.970 (2.28 ppm), both with +2 charge state, present in 1022X.S.1B.7A fractions. In the MS^2^ spectra of these compounds (Figure S4), they are differentiated from each other by their lipid side chain product ions, *m/z* 325.28 for turnercyclamycin A, and *m/z* 351.30 for turnercyclamycin B. Other minor analogues, turnercyclamycins C and D previously reported were not identified in the fractions.

The pharmacokinetics, toxicity, and efficacy of turnercyclamycin A was previously investigated [25] and showed that turnercyclamycin A exhibited reasonable in vivo pharmacokinetics parameters in an IV mouse model comparable to other lipopeptide antibiotics. An initial study of its mechanism of action also showed that it does not enter cells but is concentrated in the blood plasma. Turnercyclamycin A was also reported to have potency against colistin-resistant *Acinetobacter* complex strains [25]. The mechanism of action is still being studied. 

#### 3.1.3. Oxylipins

In the paper by Lacerna et al. [26], turneroic acid together with known oxylipins (E)-11-oxooctadec-12-enoic acid, and (E)-9-oxohexadec-10-enoic acid was purified from the culture broth of *T. turnerae* 991H.S.0A.06B. All of these compounds were observed to have biofilm inhibition activities against *Staphylococcus epidermidis* [26]. 

Sodiated precursor ion *m/z* 337.236 (1.48 ppm) from the MS^1^ of *T. turnerae* 991H.S.0A.06B fractions in this study was identified as turneroic acid. By comparison of the reported MS^2^ spectra and the experimental spectra, the precursor ion *m/z* 279.233 (4.29 ppm) was identified as (E)-11-oxooctadec-12-enoic acid (Figure S5). The nodes *m/z* 279.216 and 279.196 representing this compound belong to the biggest cluster in this molecular network generally classified as lipids (Figure S6). 

GNPS has annotations for the other precursor ions present in this cluster. However, upon manual inspection of the tail-to-tail spectral match and calculation of the mass error, these annotations did not meet the set criteria for considering a putative identification in this study. The annotations for these precursor ions are structurally diverse lipids such as steroids, alkaloids, diterpenoids, oxylipins, unsaturated fatty acids, glycerolipids, epoxide derivatives, polyketides, sesquiterpenoids, and sterols. Although the annotations are not exact, they may serve as a starting guide for the possible structures of these unknown lipids. 

#### 3.1.4. Teredinibactins

Teredinibactin A, a chlorinated compound that forms complexes with copper, iron, and molybdenum, and its dechlorinated analogue, dechloroteredinibactin, were recently reported [9]. Teredinibactin A was putatively identified in fractions of *T. turnerae* 991H.S.0A.06B as the protonated precursor ion with *m/z* 331.016 (4.23 ppm) (Figure S7). The proposed fragmentation analysis of teredinibactin A (Figure S8) was consistent with the product ions generated from the experimental data and reference literature. Another protonated precursor ion with *m*/*z* 297.055 (3.70 ppm) from the fractions of *T. turnerae* 1022X.S.1B.7A was putatively identified as dechloroteredinibactin. Based on the spectra, three diagnostic peaks can be recognized as signatures of teredinibactins: *m*/*z* 162, 144, and 116 indicating the presence of a thiazoline moiety. Two other analogous compounds were also identified within the cluster with *m*/*z* 374.964 (0.80 ppm) and 345.032 (4.06 ppm), albeit with signal intensities significantly lower than the two previous compounds (Figure S7). The isotopic distribution of product ions for *m*/*z* 374.964 indicated the presence of bromine instead of chlorine. This was also alluded to in the previous literature but was not isolated for characterization [9]. 

On the other hand, the analogue with *m/z* 345.0320 compared with teredinibactin A has a mass difference of 14.0156 suggesting the replacement of the -OH in the carboxyl group (C12) with an -OCH_3_. Fragmentation analysis (Figure S8) supports this annotation. It should be noted, however, similar to the brominated teredinibactin A, the signal intensity for this compound is low. Presently, the physiological role of teredinibactins in the *T. turnerae* is still unclear.

### 3.2. Dereplication Based on GNPS Molecular Networking 

**Figure 3:**
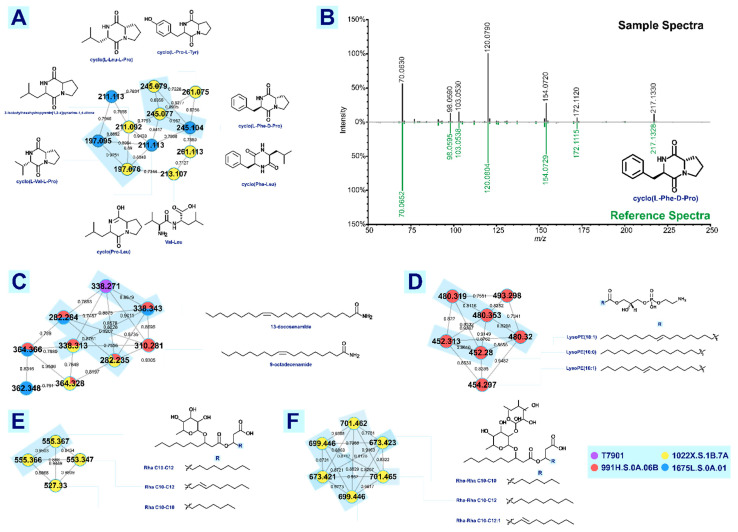
Clusters annotated from GNPS matches and verified through mass error calculation and fragmentation analysis. Figure shows the different clusters automatically annotated by GNPS with a mass error of less than 5 ppm. Cluster (**A**) is the cyclic dipeptides cluster, the cluster (**C**) is the fatty acid amide cluster, cluster (**D**) is the lysophosphatidylethanolamine or LPEs cluster, cluster (**E**) is the monorhamnolipids while Cluster (**F**) is the dirhamnolipid counterparts. (**B**) is a representative tail-to-tail match of experimental data and reference spectra from GNPS. All matches within the clusters were verified using tail-to-tail match, cosine score, and mass error. Only identifications within the mass error limit of 5 ppm were accepted as a match. Structure corresponding to the putative identifications of compounds can also be seen in the figure. Nodes that are similar in experimental spectra and identification are grouped together.

Five clusters (Figure 3) were annotated using GNPS, and mirror matching for each library hit was carefully examined to find the best match based on mass accuracy and cosine score. The list of compounds that have been found via library matching are tabulated (Table 2) and each cluster will be discussed in detail below.

#### 3.2.1. Fatty Acid Amide

A cluster of lipids were annotated as fatty acid amide with two nodes putatively identified as (13Z)-docosenamide (*m/z* 338.3416, 2.04 ppm) and (9Z)-octadecenamide, (*m/z* 282.2780, 3.90 ppm). Although this match was given by GNPS, manual inspection of spectra was still done to check if the match was true. Manual inspection of the product ions for both precursor ions were consistent with the product ion reported in the literature [27]. Manual structural analysis of these compounds also corroborated the GNPS annotation but with no distinguishing product ions due to the difference in chain length of the fatty acid amides. Neutral losses, e.g., loss of NH_3_, H_2_O, and C-C dissociation due to charge-directed fragmentation were consistent for both compounds. Compounds (13Z)-Docosenamide and (9Z)-Octadecenamide are present in the majority of the samples analyzed. They are necessary for the physiological and reproductive processes of marine organisms.

Lipids are one of the major sources of metabolic energy for any species, and they reflect the biochemical and ecological conditions of the marine environment [28]. (13Z)-Docosenamide, also known as erucamide, is a bioactive fatty acid amide that acts as a bioregulator with angiogenesis activity [29]. In a study involving the symbiosis of duckweed root, *Spirodela polyrrhiza*, and *Pseudomonas fluorescens*, (13Z)-Docosenamide appeared to stimulate two key denitrifying bacterial reductase enzymes, nitrate reductase and nitrite reductase [30,31]. In another study, Tamilmani et al. [32] reported (13Z)-Docosenamide as a product of bacteria in response to glucose addition, which might be a common metabolic process. This observation raised the possibility of a fundamental signaling process as a feedback mechanism of environmental parameters. Further studies regarding (13Z)-Docosenamide and shipworm symbionts should be investigated to determine its specific role and mechanism.

(9Z)-Octadecenamide, also known as oleamide, is a sleep-inducing lipid that was found in the cerebrospinal fluid of cats [33]. In a study involving the sponge-associated marine actinomycetes *Nocardiopsis dassonvillei* MAD08 [34], and the marine alga *Tetraselmis tetrathele* [35], (9Z)-Octadecenamide was found to have antimicrobial activity. These studies could suggest that the compound possibly contributes to the antimicrobial properties of *T. turnerae* T7901.

#### 3.2.2. Cyclic Dipeptides

GNPS annotated the cluster in Figure 3A as cyclic dipeptides, with an exception of one linear dipeptide. Fragmentation analysis of these compounds (Figure S8a,b) based on the mechanism proposed by Furtado et al. [36] showed diagnostic product ions that allow differentiation between amino groups. Cyclic dipeptides (CDPs) or 2,5-diketopiperazines are naturally produced and utilized in quorum sensing by bacteria. Quorum sensing (QS) is a process for bacterial cell-to-cell communication, which regulates various functions, some of which are the production of major virulence factors and biofilm formation [37,38]. Molecular rigidity and multiple hydrogen bonding sites of CDPs allow for strong intermolecular interactions [39,40]. Naturally occurring CDPs isolated from bacteria and other organisms exhibited various bioactivities such as antitumor, antibacterial, antivirus, antioxidant, antifungal, and others.

The experimental instrument conditions and parameters are not optimized to differentiate enantiomers. Manual inspection of enantiomeric CDPs available in the GNPS library shows that their major product ions are the same. Thus, the GNPS annotation that includes the configuration of the CDPs as tabulated in Table 2 is solely dependent on the mass spectral library matching algorithm and further analysis is needed for verification. This is important to mention because the stereochemistry of CDPs can affect their bioactivity. In particular, the annotated cyclo(Pro-Leu) (*m/z* 211.145, 2.84 ppm) exhibits antimicrobial activity. Its effectiveness varies against multiple pathogens depending on its configuration [38]. There are currently no reports on the bioactivity of cyclo(L-Phe-D-Pro) (*m/z* 245.128, 3.26 ppm) and cyclo(L-Val-L-Pro) (*m/z* 197.130, 5.07 ppm). Cyclo(L-Pro-L-Tyr) was reported to have antimicrobial activity against both gram-positive and gram-negative bacteria [41]. It also exhibited better antifungal activity than a standard fungicide amphotericin B against *Candida albicans*. In general, it was observed that mainly Pro, Arg, and Trp-based CDPs have antimicrobial activities. Figure S9 shows the structural analysis of cyclo(Pro-Leu) based on Furtado et al. [36].

#### 3.2.3. Lysophosphatidylethanolamine

Phosphatidylethanolamine is a class of phospholipids found in plasma membranes [42]. Lysophosphatidylethanolamine (LPE) is a derivative of phosphatidylethanolamine by deacylation of phospholipase A2 [43]. LPEs are present in small quantities as integral elements of membranes of animals, plants, and bacteria [44]. GNPS automatically annotated a cluster of lysophosphatidylethanolamine. Out of the seven nodes, only two compounds were putatively identified as 1-palmitoyl-2-hydroxy-sn-glycero-3-phosphoethanolamine (LysoPE(16:0); (*m/z* 454.294, 3.30 ppm) and 1-(9Z-octadecenoyl)-sn-glycero-3-phosphoethanolamine (LysoPE(18:1); (*m/z* 480.308, 1.67 ppm) (Figure S10). These LPEs were only observed in subfractions of 991H.S.0A.06B. Moreover, this is the first time the presence of LPEs has been reported in shipworm isolates.

Through manual inspection, one node that was not automatically annotated was putatively identified as LysoPE (16:1) (*m/z* 452.277, 0.44 ppm). This lysophosphatidylethanolamine was isolated and identified in a previous study with identical major product ions as *m/z* 452.2774 [43]. Slight differences in ion intensities can be attributed to the parameters and instrument conditions used to generate the experiment. Meylaers et al. [44] reported that the antimicrobial activity of rimenophenazine antibiotic agents was shown to be mediated by the release of lysophospholipids. The study showed that LysoPE(16:0) and lysophosphatidylcholine inhibited the growth of Gram-positive bacteria, *Bacillus thuringiensis*, and fungal *Saccharomyces cerevisiae*, with minimal growth inhibitory concentrations. In the same study, LysoPE (16:1) showed a stronger growth inhibiting property against the Gram-positive bacteria which implies that the difference in structure configuration of LPEs affects the activity. In another study by Farag et al. [45] and Ryu et al. [46], LPEs were tested to see their effect on the retardation of tomato leaf and tomato fruit senescence. LPEs were found to be biologically active lipids that regulate certain key processes during plant senescence and aging. This suggests that LPEs may have a role as a lipid mediator in cellular responses.

Function of LPEs in relation to *T. turnerae* is still unknown but it could possibly be involved in cellular response. Further studies are recommended.

#### 3.2.4. Rhamnolipids

One of the clusters automatically annotated by GNPS is a rhamnolipid cluster consisting of precursor ions ranging from *m/z* 673.3765 to *m/z* 701.4902. Nodes *m/z* 673 and 701 were annotated as sodiated Rha-Rha C10-C10 (*m/z* 673.377, 0.74 ppm) and Rha-Rha C10-C12 (*m/z* 701.409, 1.28 ppm), respectively. Upon inspection through comparison with Watrous et al. [47], the product ions generated from the ESI experiment were consistent with those generated with the published MALDI experiment on dirhamnolipids imaging with a slight variation in ion intensities due to the ionization method used (Figure S11A). The node with *m/z* 699 was manually annotated as sodiated Rha C10-C12:1 (*m/z* 699.395, 3.29 ppm) with unsaturation at its lipid hydrophobic chain. Furthermore, manual fragmentation analysis of the precursor ions reinforced the identification of these dirhamnose-lipids (Figure S11B). Most of the product ions were formed through remote hydrogen rearrangements.

Clusters with nodes ranging from *m/z* 527 to *m/z* 555 were also analyzed since two of the nodes, *m/z* 527.330 and *m/z* 553.347, were putatively identified by GNPS as Rha C10-C10 (*m/z* 527.318, 2.84 ppm) and Rha C10-C12 (*m/z* 555.350, 1.62 ppm). Fragmentation analysis (Figure S11B) showed that this cluster is possibly the monorhamnose-lipid counterpart of the dirhamnose-lipids discussed above. Biosurfactants are glycolipids composed of a polar part, sugar moiety, and a hydrophobic chain. Rhamnolipids (RLs) are biosurfactants commonly synthesized by *Pseudomonas* strains. RLs are composed of glycosides with rhamnose moieties and lipid moieties connected via *O*-glycosidic linkage [48]. They are surface-active metabolites with proven antimicrobial ability against *Listeria monocytogenes* [49]. Due to microbial fermentation, bacteria can synthesize a huge variety of rhamnolipid congeners. It is normal to expect variations in the chain length, fatty acid component’s degree of unsaturation, and the number of rhamnoses. The bioavailability of hydrocarbon substrate as a carbon source is reported to be improved by RLs [50]. The presence of RLs in the strains of *T. turnerae* might be due to the mechanism of how *T. turnerae* utilizes the hydrocarbon source. As of this writing, the production of rhamnolipids by *T. turnerae* has not been reported yet and is only seen in the subfraction of 1022X.S.1B.7A.

### 3.3. Dereplication Based on Manual Dissociation Analysis

#### 3.3.1. Antibiotic Precursors Erythronolide A and Nonactyl Homononactoate

A cluster (Figure S12) with masses ranging from *m/z* 363.181 to *m/z* 451.254 did not yield any annotation with GNPS library matching, classical molecular networking, and MolNetEnhancer. As an alternative, manual annotation with reference to the Dictionary of Natural Products^®^ (DNP) and the Comprehensive Marine Natural Products Database (CMNPD) [19] yielded putative identification of nodes *m/z* 441 and *m/z* 423 as erythronolide A (*m/z* 441.246, 0.68 ppm) and nonactyl homononactoate (*m/z* 423.236, 0.95 ppm), respectively.

**Figure 4:**
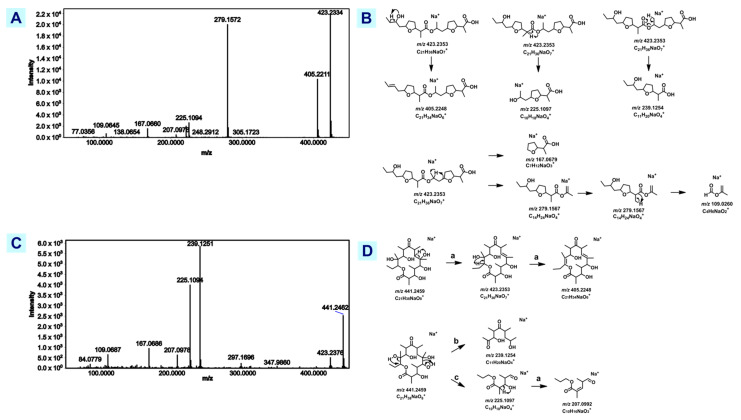
Structural analysis of nonactyl homononactoate and erythronolide A. (**A**) Experimental MS^2^ spectra of nonactyl homononactoate. (**B**) Proposed fragmentation mechanism of nonactyl homononactoate (**C**) Experimental MS^2^ spectra of erythronolide A. (**D**) Proposed fragmentation mechanism of erythronolide A.

Through a manual database search using DNP and a proposed fragmentation pathway, [M+Na]^+^ = 441.2462 was putatively identified as erythronolide A, a precursor of the macrolide antibiotic, erythromycin. This node is only present in T7901. A fragmentation scheme based on the literature is proposed in Figure 4 [51,52]. One of the BLASTp hits of an intestine-derived type I PKS contigs from the symbiotic microbiome of the mangrove shipworm *Neoteredo reynei* is an erythronolide synthase [53]. This BGC was not described in the genome of *T.turnerae* [5].

Through manual search in DNP, [M+Na]^+^ = 423.238 was putatively identified as nonactyl homononactoate or feigrisolide C. A proposed fragmentation scheme was done based on the work of Crevelin et al. [54] in Figure 4. Nonactyl homonoactoate or feigrisolide C has 8-stereocenters and is a linear dimer related to nonactin, a known macrotetrolide antibiotic isolated from marine-derived *Streptomyces* sp. This compound is reported to have antibacterial properties based on the findings by Tang et al. [55]. Samples from three strains (fractions of 991H.S.0A.06B, 1675L.S.0A.01, 1022X.S.1B.7A, and subfraction of 1022X.S.1B.7A) were found to contain *m/z* 423.16, which could possibly suggest a contribution to the significant activity of the samples since subfractions of 991H.S.0A.06B and 1022X.S.1B.7A are active against *S. aureus* and MRSA (methicillin-resistant *S. aureus*). However, spectral comparison with Crevelin et al. [54] showed a difference in one of the product ions. There is a high intensity for *m/z* 239 as published by Crevelin et al. [54] but was absent in the experimental spectra. This may be due to the difference in experimental setup requiring further verification.

#### 3.3.2. Lyso-Ornithine Lipids

**Figure 5:**
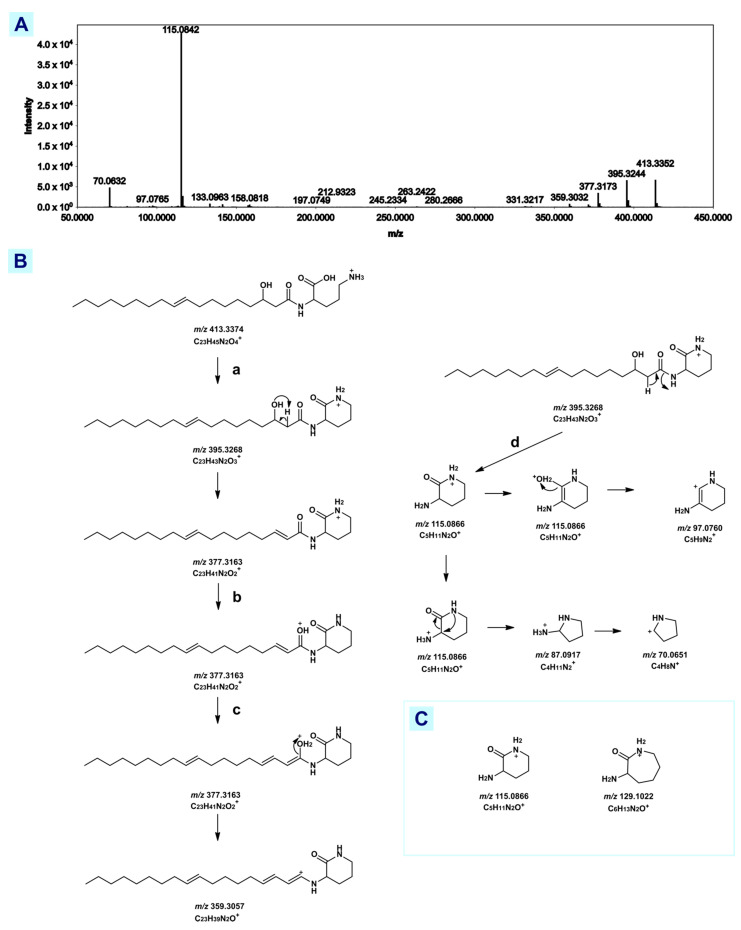
Structural analysis lyso-ornithine lipids. (**A**) Experimental MS^2^ spectra of *m/z* 413.333 (**B**) proposed fragmentation pathway of lyso-ornithine lipid *m/z* 413.333 (**C**) Characteristic product ions present in all lyso-ornithine lipid within the cluster.

GNPS annotated nodes *m/z* 413.333, 413.317, and 414.336 as ‘putative (3-hydroxyheptadecanoyl)lysine’ while *m/z* 401.305 was annotated as ‘putative (3-hydroxyhexadecanoyl)lysine’ (Figures S13 and S14). Closer inspection of a representative spectrum revealed that *m/z* 115.08 is highly abundant (Figure 5A) instead of *m/z* 129.10, a cyclic product ion for lysine lipids [56,57]. It has been reported that *m/z* 115.08 is a diagnostic product ion for cyclic ornithine lipids [57,58]. A theoretical fragmentation scheme for lyso-ornithine lipids (LOLs) is hereby presented (Figure 5B) showing the formation of the 3-amino-2-oxopiperidinium ion (*m/z* 115.08) and protonated ornithine (*m/z* 133.09). These relevant results support the annotation of this cluster as lyso-ornithine instead of lyso-lysine lipid.

Reported MS^2^ of C_23_H_44_N_2_O_4_, a LOL that acts as a biosurfactant produced by *E. coli* (EC-ORF32) and *Pseudomonas putida* (PP54F3) [59], was generated with a UHPLC-HESI-Quad-Orbitrap (1290 UHPLC System Agilent, HESI Q-Exactive Plus Orbitrap MS ThermoFischer). Using these reference spectra corroborated with a fragmentation analysis, *m/z* 413.339 (4.60 ppm) (Figure 5A,B) is putatively identified as C_23_H_44_N_2_O_4_ LOL. Though there was an observed difference in ion intensities, variation is accounted for by the difference in ion source (HESI vs. ESI).

Another report has identified and characterized C_21_H_42_N_2_O_4_ was generated with an orbitrap mass spectrometer (Thermo Scientific™ Orbitrap ID-X™ Tribrid™ Mass Spectrometer), generating a fragmentation pattern that matches the experimental spectra of *m/z* 387.323 (2.58 ppm) (Figure S14) [59]. This compound is a new iso-branched LOL from an Arctic marine bacterium, *Lacinutrix sp*. that exhibited cytotoxic activity against A2058 human melanoma cells [60,61]. Production of LOLs by *T. turnerae* has not yet been reported. Since these LOLs were only putatively identified through a comparison of MS^2^ data available in the literature from other bacterial species, further structural analysis of this group of compounds in *T. turnerae* is needed for verification. Other possible unreported LOL analogues or related compounds may be identified in the future given the size of the cluster.

Shown in Table 2 is the summary of all compounds annotated from the literature, GNPS, and manual dereplication. This shows the diversity in small molecules that can be found in shipworm endosymbiont strains. Fatty acid amides, cyclic dipeptides, rhamnolipids, lysophosphatidylethanolamines, tartrolons, turnercyclamycins, teredinibactins, oxylipins, antimicrobial precursor compounds, and lyso-ornithine lipids—which could have contributed to the bioactivity of the strains based on the literature. Some of the metabolites are unique to a specific strain, i.e., turnercyclamycins and rhamnolipids in 1022X.S.1B.7A, and oxylipins in 991H.S.0A.06B, while some are found across all strains. Potential analogues of the cytotoxic macrodiolide tartrolon D and the metal-binding teredinibactin A are yet to be fully characterized. Biofilm-inhibiting oxylipin, 11-oxooctadec-12-enoic acid, belongs to the biggest cluster of unannotated lipids. Major constraints annotation with metabolomics, in this case, is the limited database information available on metabolites produced by microorganisms, especially from marine sources. Structural motifs from product ions (data not included) were also useful for annotation, especially for analogous compounds but within the bounds of the database deposits. Theoretical fragmentation analysis also provided structural details, helping reinforce the putative identification of compounds. Molecular networking and theoretical structural fragmentation were shown to be valuable as tools for dereplication.

## 4. Conclusions

Small molecule profiling using UPLC-MS/MS enabled the bioprospecting of *T. turnerae*, the intracellular endosymbiont of shipworms sampled from Bohol and Sultan Kudarat, Philippines. Molecular networking showed the chemical space of the three strains investigated with more than 93 clusters of two or more nodes.

This work also recognised that cosine score alone cannot give the full relationship of nodes in molecular networking and mirror matching in library search. The cosine score was designed to describe spectral similarity, i.e., mirror matching of precursor *m/z* and intensity. This technique is widely used in joining thousands of spectra into one consensus spectrum and relating the nodes in molecular networking. However, in this analysis, spectral similarity scoring was not sufficient enough to fully unravel the information presented by the data, as seen by the number of clusters that were not annotated. The crux of metabolomics annotation through molecular networking lies within the similarity of structures. In the study by Huber et al. [19]., a new similarity score algorithm was laid out in Spec2Vec, which scores the similarity of spectra based on related fragments and losses instead of the precursor *m/z* and intensities. Spec2Vec has demonstrated to increase the number of true matches compared to spectral similarity scoring. To locate true matches or spectra belonging to the same chemical class, Spec2Vec will be helpful in future GNPS analysis.

Overall, metabolomics studies via LC-MS enabled the putative identification of metabolites produced by cultured shipworm endosymbionts. Molecular networking revealed a vast chemical space that includes clusters of known *T. turnerae* bioactive compounds. Although only a small portion of the chemical space was accounted for, this work nonetheless exhibited that shipworm endosymbionts are capable of producing a diverse array of potentially bioactive small molecules, which remain to be characterized.

## Figures and Tables

**Table 1:** Details on the *T. turnerae* strains isolated from shipworms.

Isolate Code	Sampling Site	Date Collected	Host Organism	Part
991H.S.0A.06B	Brgy Danao, Panglao Bohol Philippines	4 May 2010	* Lyrodus pedicellatus *	gill/ctenidium
1675L.S.0A.01	Brgy Sta Ana, Kalamansig, Sultan Kudarat Philippines	24 November 2010	* Kuphus polythamia *	gill/ctenidium
1022X.S.1B.7A	Cataban; Camotes Sea, Talibon, Bohol Philippines	1 May 2010	* Lyrodus massa *	caecum
T7901 (reference strain)	Newport Estuary, Beaufort North Carolina, USA	1979 by John Waterbury	* Bankia gouldi *	gill/ctenidium

**Table 2:** List of annotated metabolites from priority strains.

Compound Name	Major Ion	Monoisotopic Mass	Accurate Mass	Mass Error (ppm)	Cosine Score	Strains
Tartrolon D	[M+Na]^+^	843.450	843.451	1.19	MN/FA *	T7901, 991H.S.0A.06B, 1022X.S.1B.7A
Turnercyclamycin A	[M+H]^+^	786.958	786.955	3.18	MN/FA	1022X.S.1B.7A
Turnercyclamycin B	[M+H]^+^	799.966	799.969	4.13	MN/FA	1022X.S.1B.7A
Teredinibactin A	[M+H]^+^	331.016	331.016	4.23	MN/FA	991H.S.0A.06B, 1022X.S.1B.7A
Dechloroteredinibactin	[M+H]^+^	297.055	297.055	3.37	MN/FA	1022X.S.1B.7A
Brominated teredinibactin	[M+H]^+^	374.964	374.964	0.80	MN/FA	1022X.S.1B.7A
Teredinibactin A + (CH_3_)	[M+H]^+^	345.031	345.032	4.06	MN/FA	1022X.S.1B.7A
Turneroic Acid	[M+Na]^+^	337.235	337.236	1.48	MN/FA	991H.S.0A.06B
11-oxooctadec-12-enoic acid	[M+H-H_2_O]^+^	279.232	279.233	2.86	MN/FA	991H.S.0A.06B
(13Z)-docosenamide	[M+H]^+^	338.342	338.342	0.30	0.81	T7901, 991H.S.0A.06B, 1022X.S.1B.7A, 1675L.S.0A.01
(9Z)-octadecenamide	[M+H]^+^	282.279	282.278	3.90	0.87	991H.S.0A.06B, 1022X.S.1B.7A, 1675L.S.0A.01
cyclo(L-Val-L-Pro)	[M+H]^+^	197.128	197.129	5.07	0.88	1022X.S.1B.7A, 1675L.S.0A.01
cyclo(L-Phe-D-Pro)	[M+H]^+^	245.128	245.128	3.26	0.93	1022X.S.1B.7A, 1675L.S.0A.01
cyclo(Pro-Leu)	[M+H]^+^	211.144	211.145	2.84	0.87	1675L.S.0A.01
3-(2-methylpropyl)-2,3,6,7,8,8a-hexahydropyrrolo[1,2-a]pyrazine-1,4-dione	[M+H]^+^	211.144	211.145	2.84	0.83	1022X.S.1B.7A
Val-Leu	[M+H-H_2_O]^+^	213.156	213.159	1.88	0.86	1022X.S.1B.7A
1-palmitoyl-2-hydroxy-sn-glycero-3-phosphoethanolamine	[M+H]^+^	454.293	454.294	3.30	0.94	991H.S.0A.06B
1-(9Z-octadecenoyl)-sn-glycero-3-phosphoethanolamine	[M+H]^+^	480.309	480.308	1.67	0.88	991H.S.0A.06B
LysoPE (16:1)	[M+H]^+^	452.277	452.277	0.44	MN/FA	991H.S.0A.06B
Rha Rha C10-C10	[M+Na]^+^	673.377	673.377	0.74	0.87	1022X.S.1B.7A
Rha Rha C10-C12:1	[M+Na]^+^	699.393	699.395	3.29	MN/FA	1022X.S.1B.7A
Rha Rha C10-C12	[M+Na]^+^	701.408	701.409	1.28	0.75	1022X.S.1B.7A
Rha C10-C10	[M+Na]^+^	527.319	527.318	2.84	MN/FA	1022X.S.1B.7A
Rha C10-C12:1	[M+Na]^+^	553.335	553.336	2.17	MN/FA	1022X.S.1B.7A
Rha C10-C12	[M+Na]^+^	555.350	555.349	1.62	MN/FA	1022X.S.1B.7A
Erythronolide A	[M+Na]^+^	441.246	441.246	0.68	MN/FA	T7901
Nonactyl homononactoate	[M+Na]^+^	423.235	423.236	0.95	MN/FA	991H.S.0A.06B, 1022X.S.1B.7A, 1675L.S.0A.01
C_21_H_42_N_2_O_4_	[M+H]^+^	387.322	387.323	2.58	MN/FA	991H.S.0A.06B, 1022X.S.1B.7A, 1675L.S.0A.01
C_23_H_44_N_2_O_4_	[M+H]^+^	413.337	413.339	4.60	MN/FA	T7901, 991H.S.0A.06B, 1022X.S.1B.7A, 1675L.S.0A.01

* MN/FA = manual annotation/fragmentation analysis.

## Data Availability

All relevant data are within the paper and supplementary information file. In addition, the compiled MS data file will be uploaded to MassIVE upon publication of this work.

## Supplementary Materials

The following supporting information can be downloaded at: https://www.mdpi.com/article/10.3390/metabo12111152/s1, Figure S1: Bioactivity profiles of crude ethyl acetate extracts and partitioned fractions from different *T. turnerae* strains against different pathogens; Figure S2: Fragmentation analysis of tartrolon D; Figure S3: MS^2^ spectra of tartrolon D and its possible analogues; Figure S4: MS^2^ spectra of turnercyclamycin A and B; Figure S5: MS^2^ spectra of turneroic acid and MS^2^ spectra of (E)-11-oxooctadec-12-enoic acid; Figure S6: Lipids cluster; Figure S7: MS^2^ spectra of teredinibactins; Figure S8: Fragmentation analysis of teredinibactin A; Figure S9: Structural analysis of diketopiperazines; Figure S10: Structural analysis of lysophosphatidylethanolamines; Figure S11: Structural analysis of rhamnolipids; Figure S12: Clusters manually annotated through a database search and fragmentation analysis; Figure S13: Tail-to-tail alignments for putative (3-hydroxyhexadecanoyl)lysine and (3-hydroxylheptadecenoyl)lysine; Figure S14: MS^2^ spectra of lyso-ornithine lipid; Table S1: Description of the 35 samples included in the molecular network.

## Abbreviations

LC-MS	Liquid Chromatography-Mass Spectrometry
UHPLC-HRMS-QTOF	Ultrahigh-performance liquid chromatography with high-resolution mass spectrometry quadrupole time-of-flight
GNPS	Global Natural Products Social molecular networking
DNP	Dictionary of Natural Products^®^
CMNPD	Comprehensive Marine Natural Products Database
LPEs	lysophosphatidylethanolamine
CDPs	cyclic dipeptides
QS	quorum sensing
ESI	Electrospray Ionization
MALDI	Matrix-assisted laser desorption/ionization
RLs	Rhamnolipids
PKS	Polyketide synthase
LOLs	Lyso-ornithine lipids
SBM	Shipworm basal medium
OCF	Open column fraction
CID	Collision-induced dissociation
RP-HPLC	Reversed-phase High Performance Liquid Chromatography
MeOH	Methanol
H_2_O	Water
CH_3_CN	Acetonitrile
BGC	Biosynthetic Gene Cluster
