# Supplementary material for: Mining Small Molecules from Teredinibacter turnerae Strains Isolated from Philippine Teredinidae
Source: Metabolites. 2022 Nov 21;12(11):1152. doi: 10.3390/metabo12111152 (PMC9692312; doi:10.3390/metabo12111152)
Supplement: Supplementary file 1 [file metabolites-12-01152-s001.zip › metabolites-2018315-supplementary.pdf]

Supplementary Material

Mining Small Molecules from *Teredinibacter turnerae* strains isolated from Philippine Teredinidae

Jamaine B. Villacorta[1], Camille V. Rodriguez[1], Jacquelyn E. Peran[2], Jeremiah D. Batucan[2], Gisela P. Concepcion[2], Lilibeth A. Salvador-Reyes[2], and Hiyas A. Junio[1,*]

[1] Small Molecules Profiling Laboratory (SMPL), Institute of Chemistry, College of Science, University of the Philippines, Diliman, Quezon City 1101, Philippines
[2] Marine Science Institute, College of Science, University of the Philippines, Diliman, Quezon City 1101, Philippines
* Correspondence: hajunio@up.edu.ph

Table of Contents

Figure S1. Bioactivity profiles of crude ethyl acetate extracts and partitioned fractions from different *T. turnerae* strains against different pathogens ………………………….…………..…..2

Table S1. Description of the 35 samples included in the molecular network…………………………. 3

Figure S2. Fragmentation analysis of tartrolon D……………………………………......…………..4

Figure S3. MS² spectra of tartrolon D and its possible analogues & Figure S4. MS² spectra of turnercyclamycin A and B………………………………………………………………………… 5

Figure S5. MS² spectra of turneroic acid and MS² spectra of (E)-11-oxooctadec-12-enoic acid ………6

Figure S6. Lipids cluster…………………………………………………………………….……….7

Figure S7. MS² spectra of teredinibactins ……………………………………………………...………..8

Figure S8. Fragmentation analysis of teredinibactin A…………………………………………………...9

Figure S9. Structural analysis of diketopiperazines……………………………………..………….10

Figure S10 Structural analysis of lysophosphatidylethanolamines ……………………………………11

Figure S11. Structural analysis of rhamnolipids & Figure S12. Clusters manually annotated through a database search and fragmentation analysis……………………………………………………..12

Figure S13 Tail-to-tail alignments for putative (3-hydroxyhexadecanoyl)lysine and (3-hydroxylheptadecenoyl)lysine & Figure S14. MS² spectra of lyso-ornithine lipid……….………13

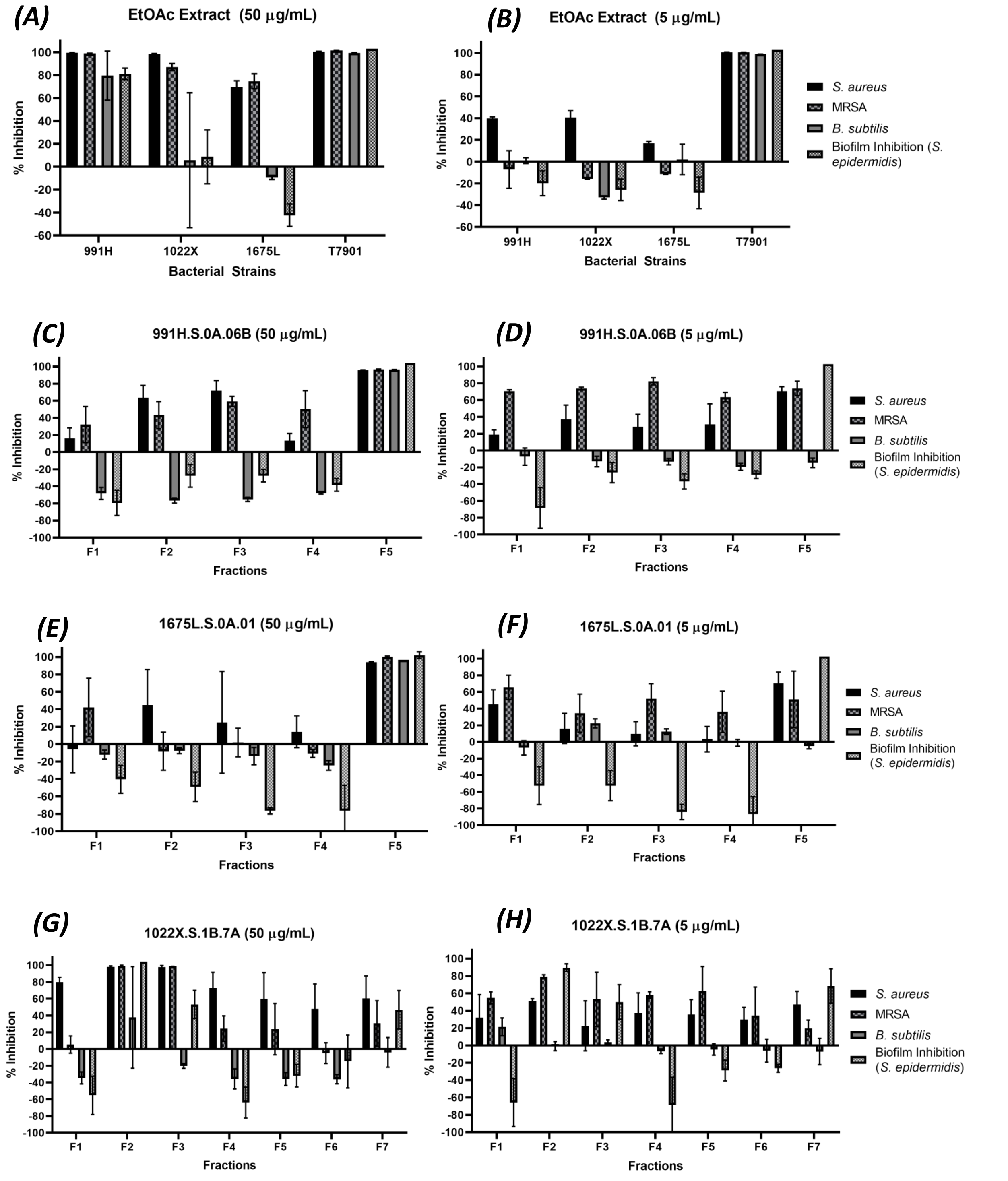

Figure S1. **Bioactivity profiles of crude ethyl acetate extracts (a-b) and partitioned fractions (c-f) from different *T. turnerae* strains against different pathogens.** Alexa-fluor™ WGA 488 microdilution assay was used for *S. epidermidis* ATCC® 35984™ biofilm, while Resazurin based-microdilution assays were performed in *S. aureus* ATCC® 6538™, Methicillin-resistant *S. aureus* ATCC® 43300, *and B*. subtilis ATCC® 6051™.

Table S1. Description of the 35 samples included in the molecular network

Sample name	Description	Sample name	Description
991H-F1	Fraction of 991H.S.0A.06B	1022X-F1	Fraction of 1022X.S.1B.7A
991H-F2	Fraction of 991H.S.0A.06B	1022X-F2	Fraction of 1022X.S.1B.7A
991H-F3	Fraction of 991H.S.0A.06B	1022X-F3	Fraction of 1022X.S.1B.7A
991H-F4	Fraction of 991H.S.0A.06B	1022X-F4	Fraction of 1022X.S.1B.7A
991H-F5	Fraction of 991H.S.0A.06B	1022X-F5	Fraction of 1022X.S.1B.7A
991H-F5-SF1	Subfraction of 991H-F5	1022X-F6	Fraction of 1022X.S.1B.7A
991H-F5-SF2	Subfraction of 991H-F5	1022X-F2-SF1	Subfraction of 1022X-F2
991H-F5-SF3	Subfraction of 991H-F5	1022X-F2-SF2	Subfraction of 1022X-F2
991H-F5-SF4	Subfraction of 991H-F5	1022X-F2-SF3	Subfraction of 1022X-F2
991H-F5-SF5	Subfraction of 991H-F5	1022X-F2-SF4	Subfraction of 1022X-F2
991H-F5-SF6	Subfraction of 991H-F5	1022X-F2-SF5	Subfraction of 1022X-F2
991H-F5-SF7	Subfraction of 991H-F5	1022X-F2-SF6	Subfraction of 1022X-F2
991H-F5-SF8	Subfraction of 991H-F5	1022X-F2-SF7	Subfraction of 1022X-F2
991H-F5-SF9	Subfraction of 991H-F5	1022X-F2-SF8	Subfraction of 1022X-F2
1675L-F1	Fraction of 1675L.S.0A.01	1022X-F2-SF10	Subfraction of 1022X-F2
1675L-F2	Fraction of 1675L.S.0A.01	T7901-C1	Crude extract of T7901
1675L-F3	Fraction of 1675L.S.0A.01		
1675L-F4	Fraction of 1675L.S.0A.01		
1675L-F5	Fraction of 1675L.S.0A.01		

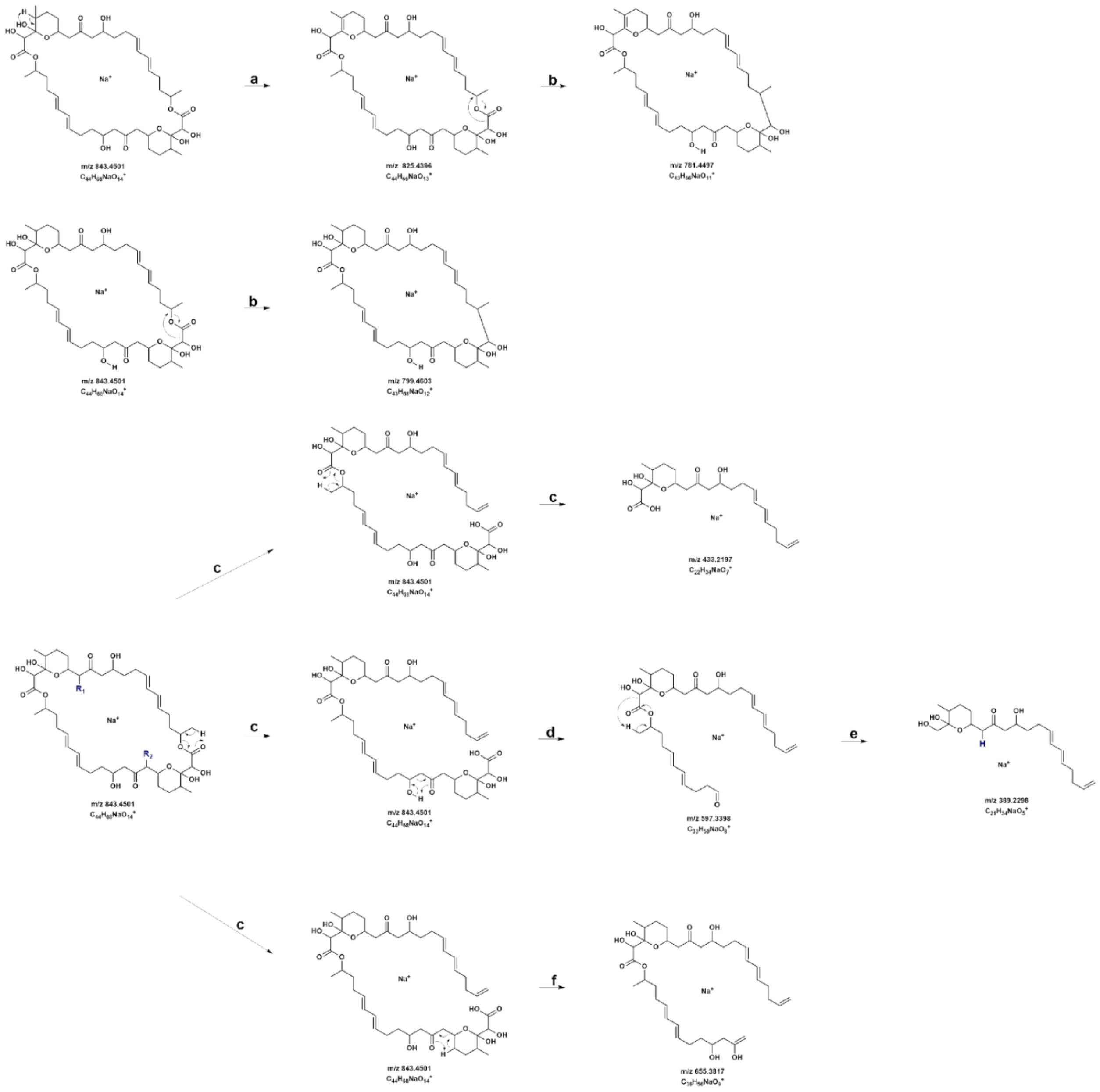

Figure S2. Fragmentation analysis of tartrolon D. For tartrolon D R_1, R_2 = H a. Loss of H_2O through remote hydrogen rearrangement b. Loss of CO_2 c. McLafferty-type rearrangement d. Retro-aldol fragmentation

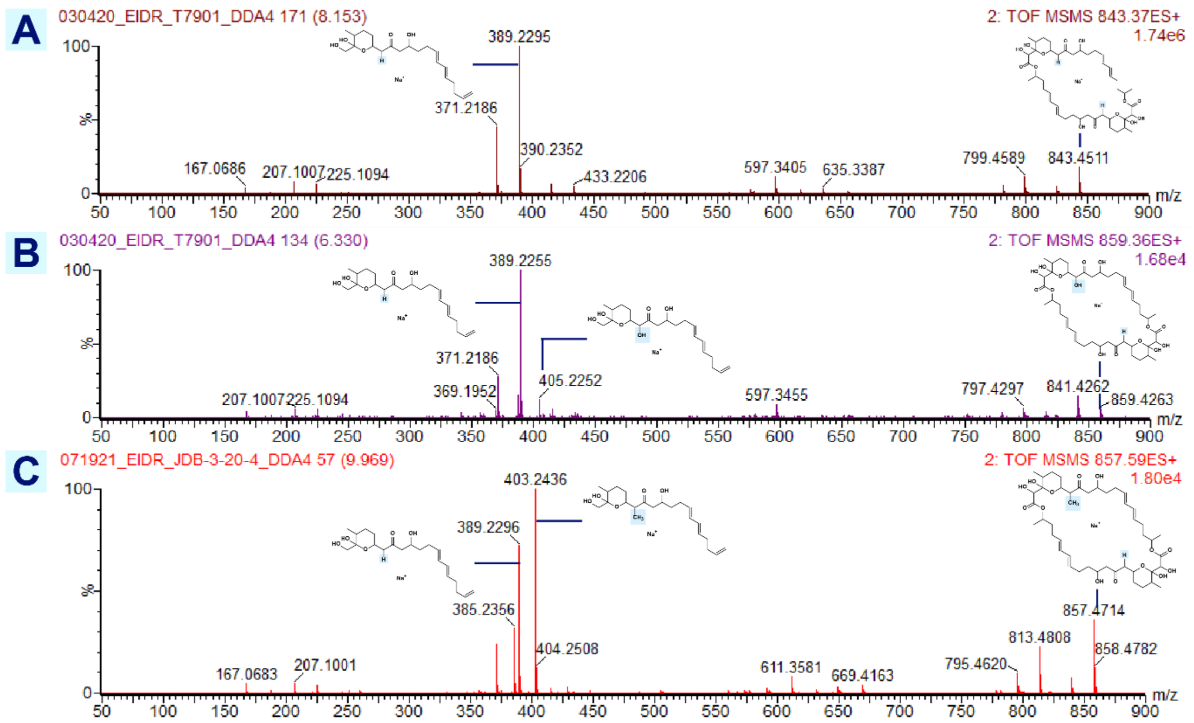

Figure S3. MS² spectra of tartrolon D and its possible analogues. A) tartrolon D B) tartrolon D analogue 1 R_1/R_2 =OH C) tartrolon D analogue 2 R_1/R_2 = CH_3

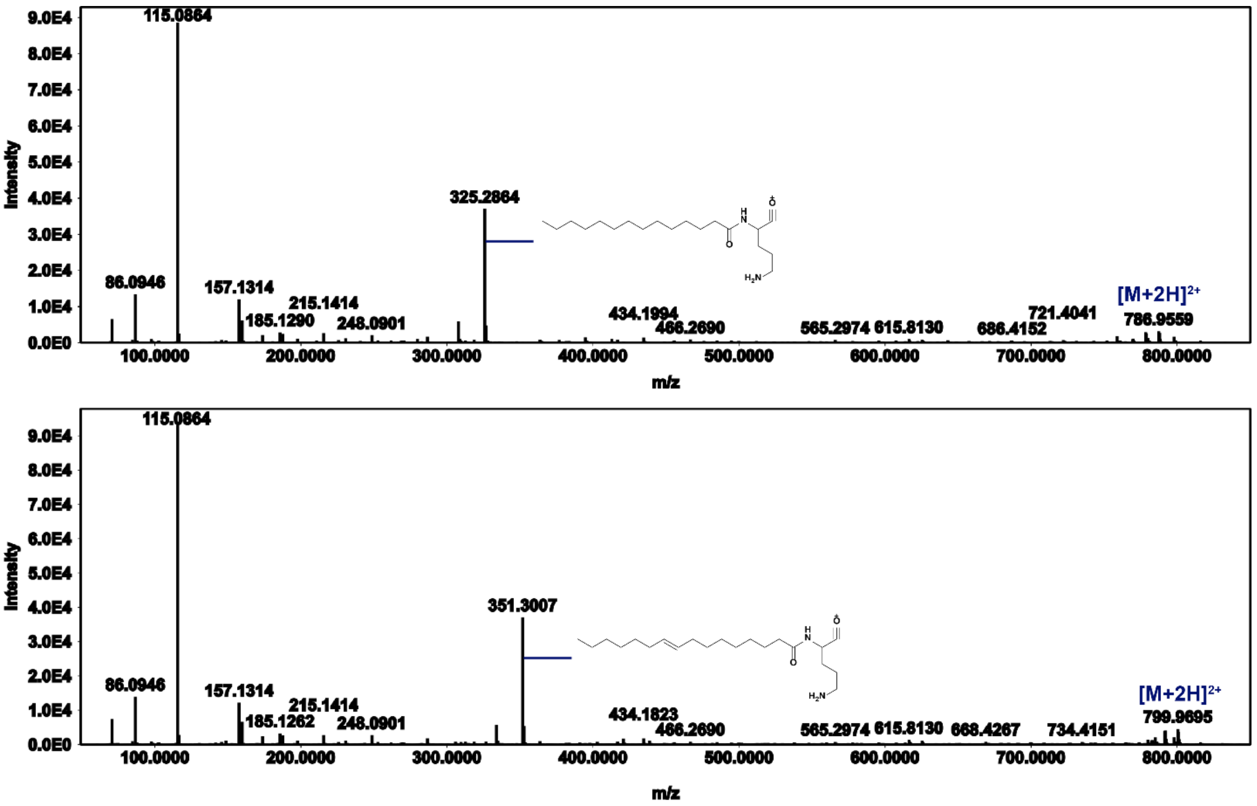

Figure S4. MS² spectra of turnercyclamycin A (top) and B (bottom). Product ions *m/z* 325.286 and *m/z* 351.3007 represent the lipid side chain of a turnercyclamycin.

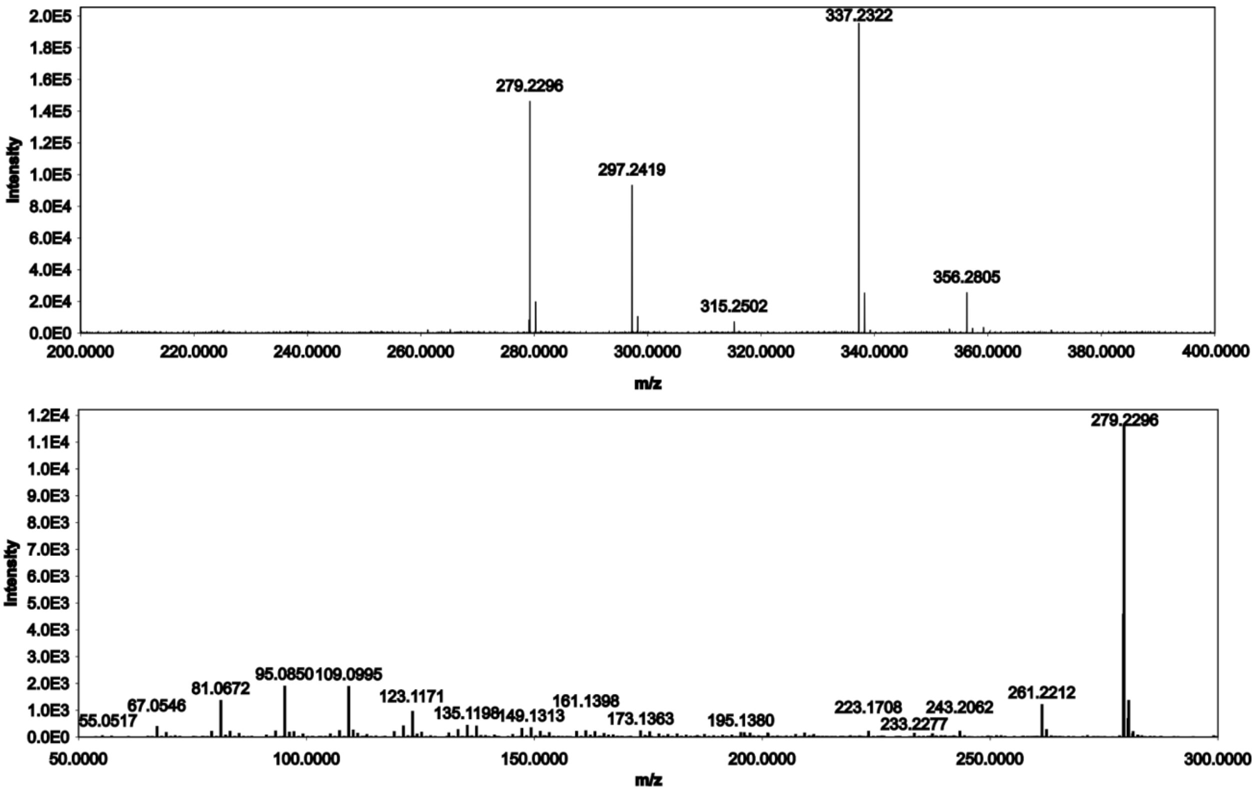

Figure S5. MS[1] spectra of turneroic acid (top) and MS[2] spectra of (E)-11-oxooctadec-12-enoic acid (bottom). Comparison with reference spectra by Lacerna et al., 2020, $[M+Na]^+$ = 337.232 and $[M+H]^+$ = 279.229 was putatively identified as turneroic acid and (E)-11-oxooctadec-12-enoic respectively.

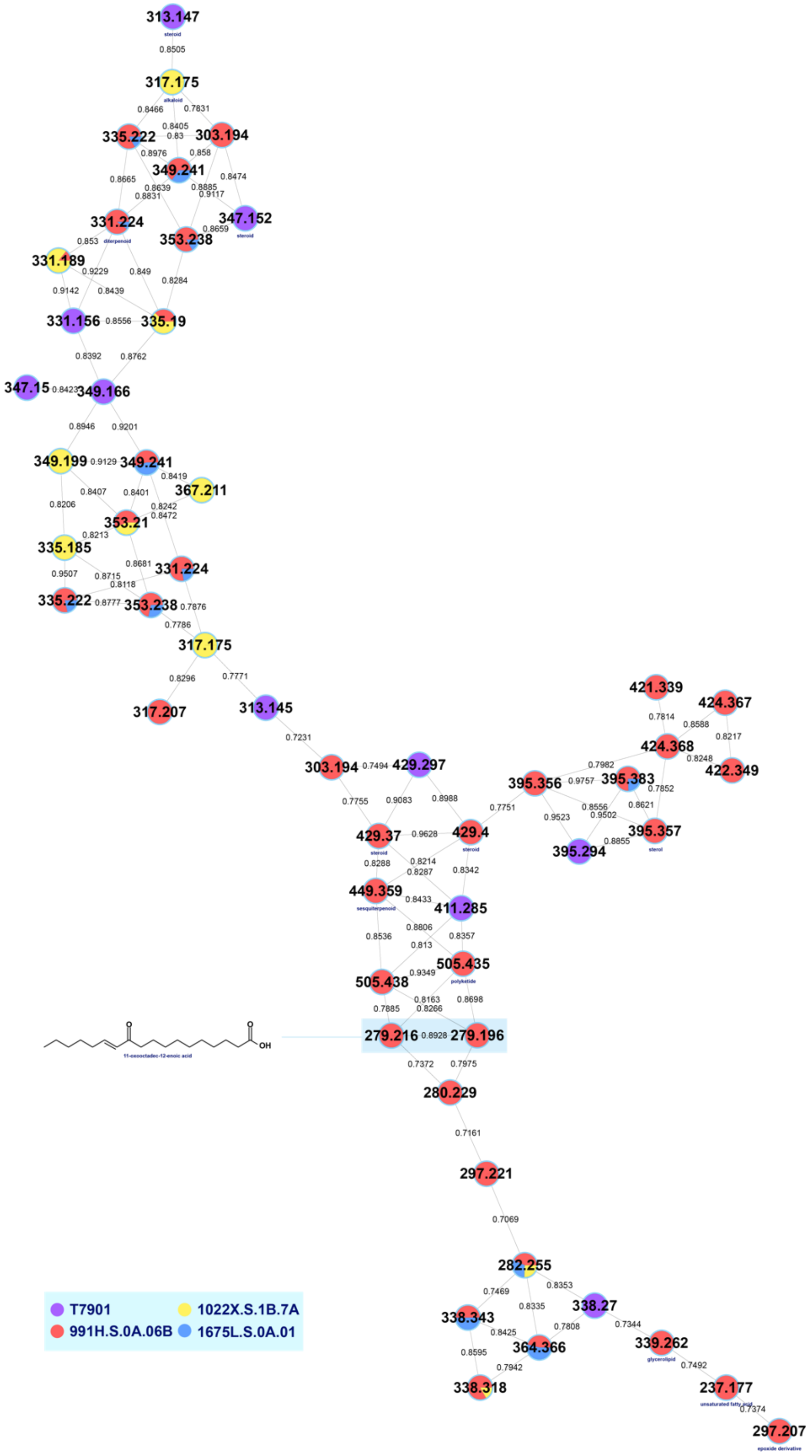

Figure S6. Lipids cluster. Out of the 52 nodes in the cluster, only *m/z* 279.216 and *m/z* 279.196 was putatively identified. Some nodes were automatically annotated by GNPS, but upon manual verification the matches were not considered. Still, these nodes are labeled by their general classification.

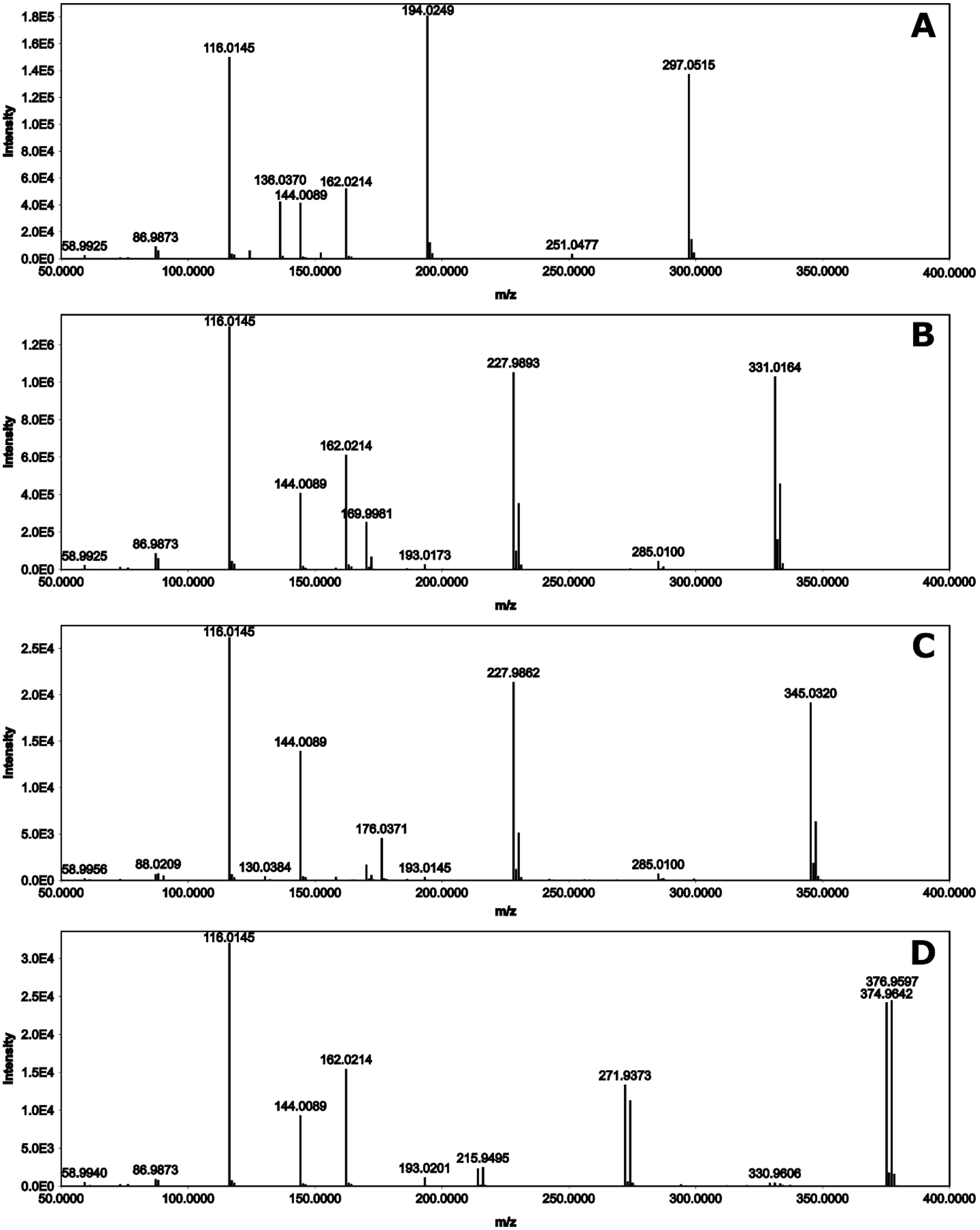

Figure S7. MS² spectra of teredinibactins. A) dechloroteredinibactin A B) teredinibactin A C) teredinibactin A with an extra methyl group D) brominated teredinibactin

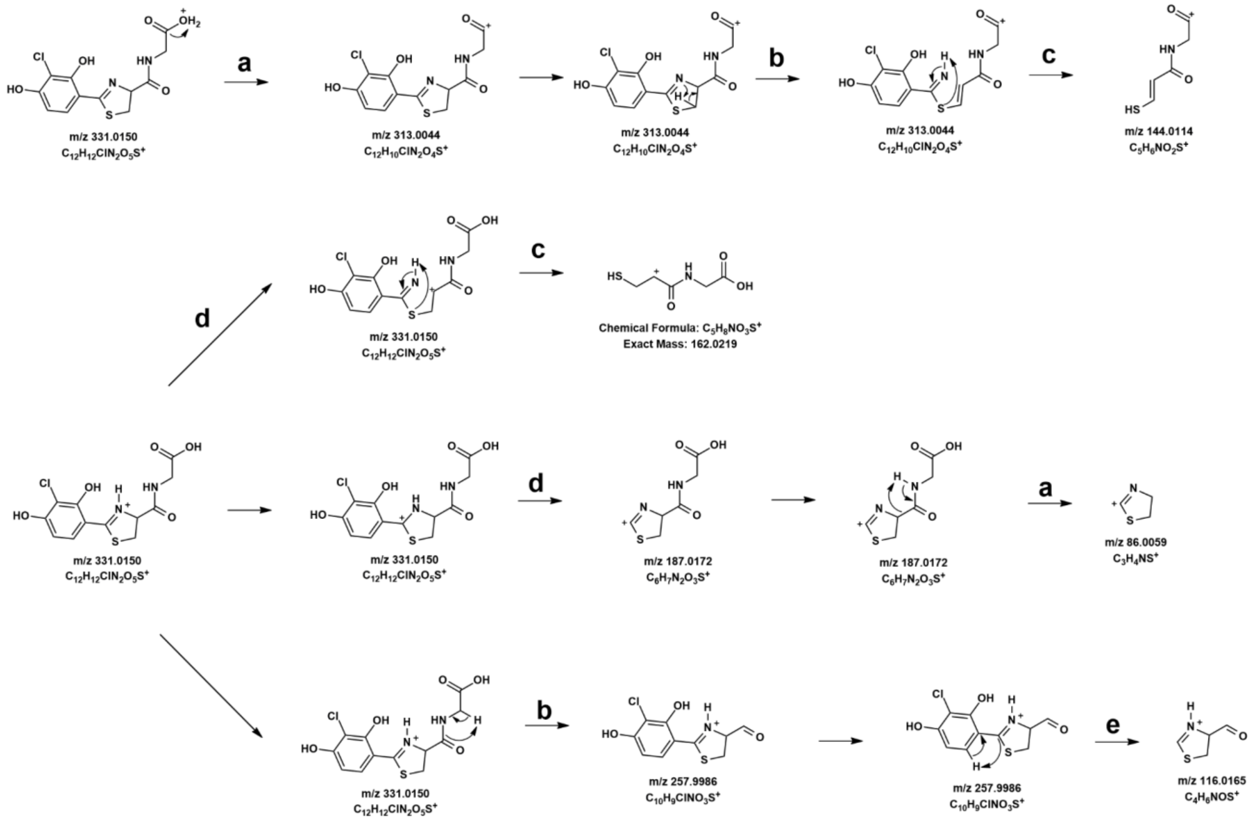

Figure S8. Fragmentation analysis of teredinibactin A. a) loss of water via inductive cleavage b) remote hydrogen rearrangement c) remote hydrogen rearrangement, formation of nitrile leaving group d) electron transfer, carbocation transfer e) formation of chlororesorcinol

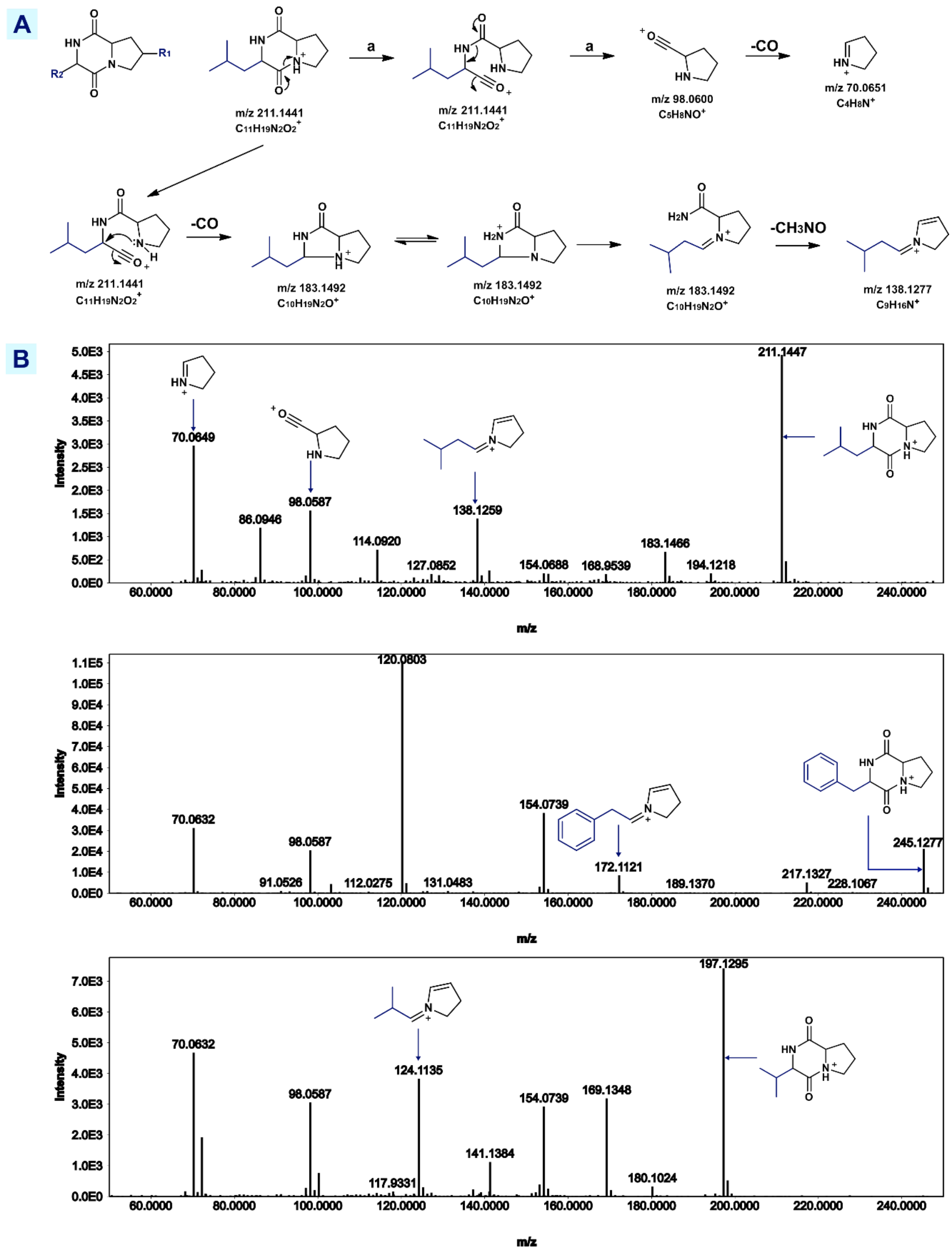

Figure S9. Structural analysis of diketopiperazines. A) Fragmentation of a representative diketopiperazine cyclo(Pro-Leu) based on the fragmentation mechanism proposed by Furtado et al. a. heterolytic cleavage b. Grob-Wharton fragmentation and c. proton migration B) From top to bottom, MS[2]

spectra of cyclo(Pro-Leu), cyclo(Phe-Pro) and cyclo(Val-Pro) with annotations of diagnostic product ions peaks.

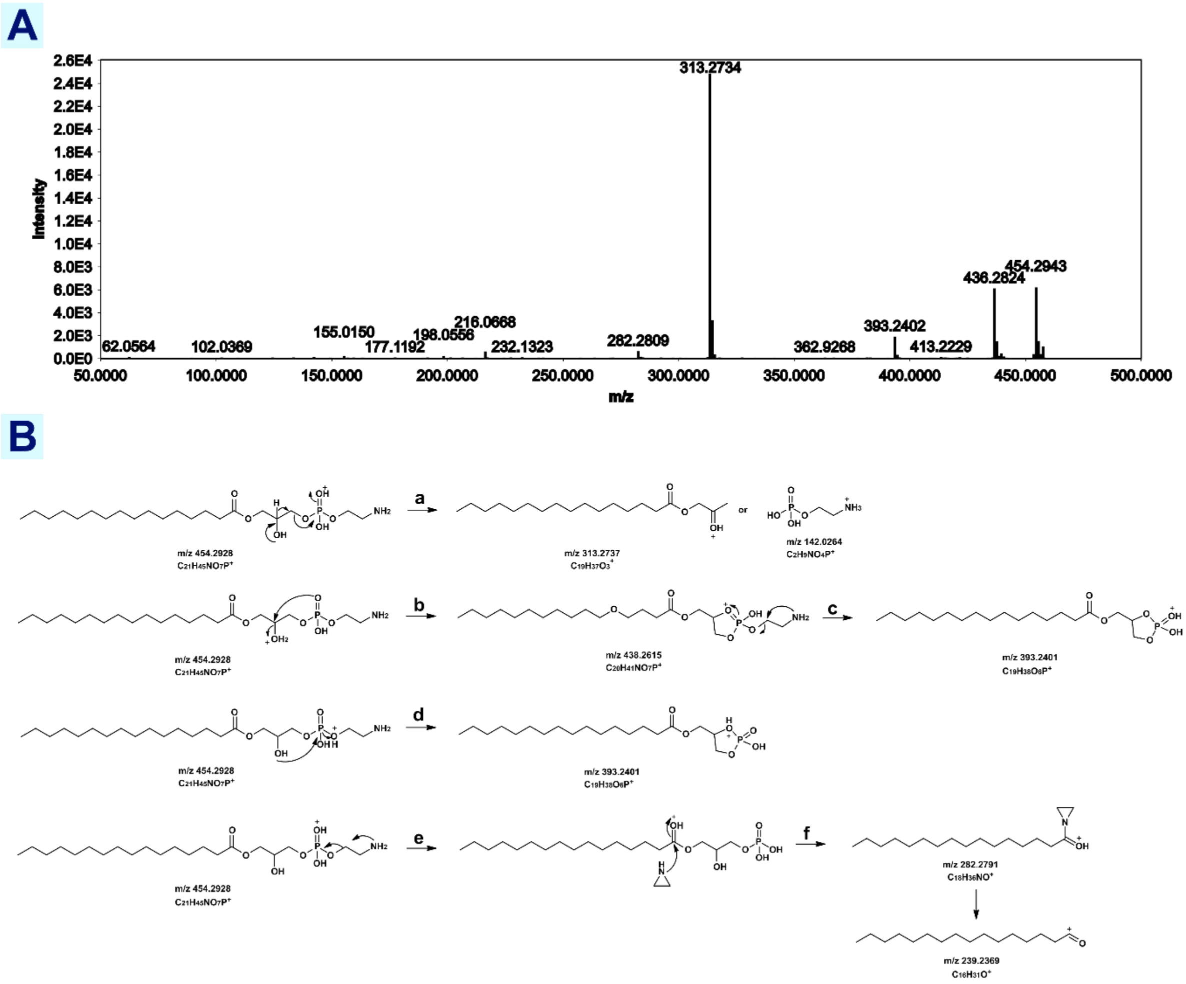

Figure S10. Structural analysis of lysophosphatidylethanolamines. A) MS^2 spectra of Rha Rha C10-C12:1 B) Fragmentation analysis of Rha Rha C10-C12:1

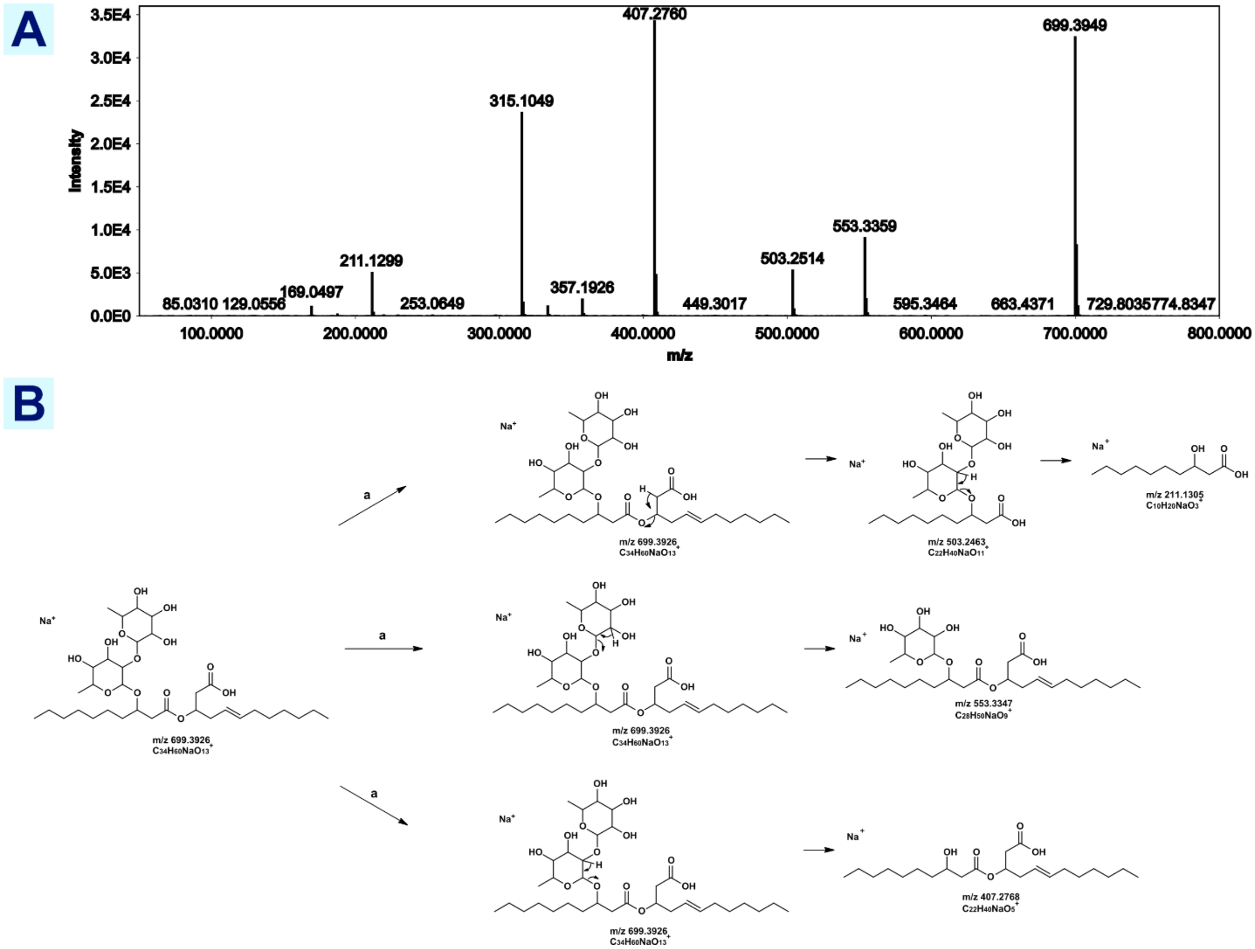

Figure S11. Structural analysis of rhamnolipids. A) MS² spectra of Rha Rha C10-C12:1 B) Fragmentation analysis of Rha Rha C10-C12:1

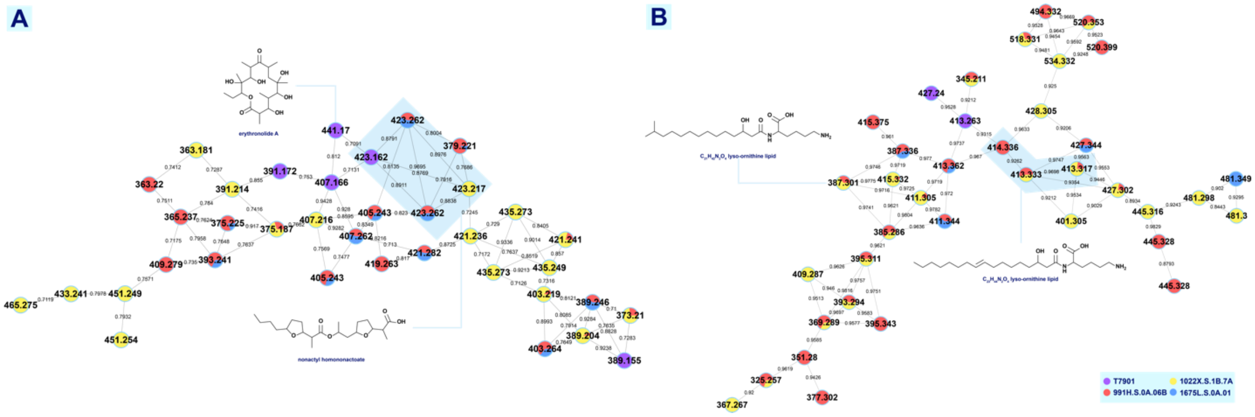

Figure S12. Clusters manually annotated through a database search and fragmentation analysis. A) A cluster containing erythronolide A and nonactyl homononactoate B) lyso-ornithine lipids

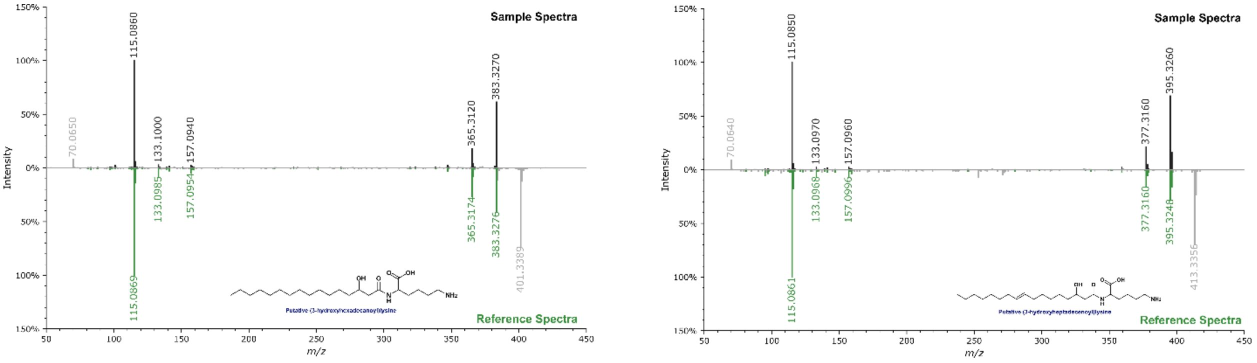

Figure S13. Tail-to-tail alignments for putative (3-hydroxyhexadecanoyl)lysine and (3-hydroxylheptadecenoyl)lysine. Most of the product ions of the sample spectra for *m/z* 383.327 and *m/z* 395.326 matched with the library spectra of putative hydroxyl lysine. From the fragmentation mechanism proposed for lyso-lysine lipids the indicative product ion *m/z* 129.1022 is absent. Thus, it is proposed that instead of a lyso-lysine lipid, these compounds are lyso-ornithine lipids based on the fragmentation analysis (Fig. 5) and the presence of the *m/z* 115.085 product ion.

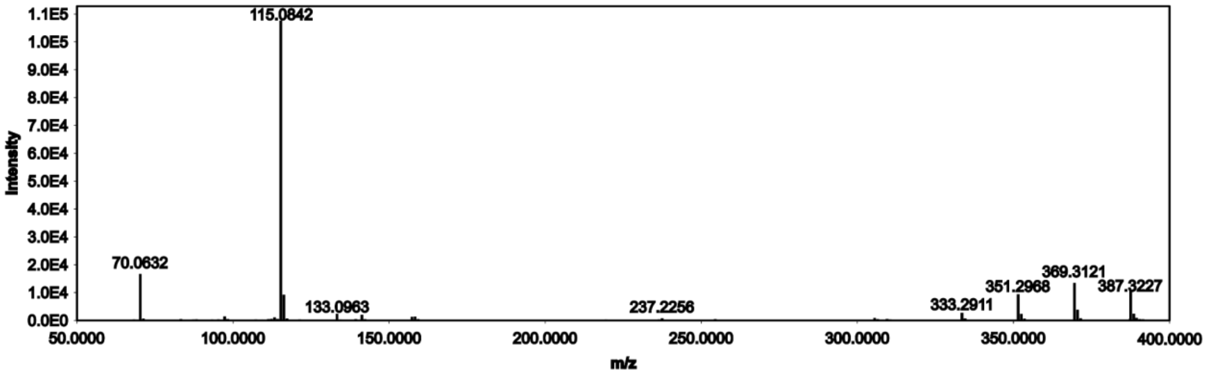

Figure S14. MS^2 spectra of lyso-ornithine lipid [M+H]$^+$ = 387.322. This MS^2 spectra was obtained at 15V-30V collision energy.
